# Development of the first European *Xanthomonas euvesicatoria* pv. *euvesicatoria* lytic bacteriophage cocktail effective in controlling bacterial spot disease in pepper plants

**DOI:** 10.3389/fmicb.2026.1821339

**Published:** 2026-05-07

**Authors:** Elena G. Biosca, Isabel Salas-Lastres, José F. Català-Senent, Félix Morán, Ana Palacio-Bielsa, Belén Álvarez

**Affiliations:** 1 Departamento de Microbiología y Ecología, Universitat de València, Valencia, Spain; 2 Departamento de Sistemas Agrícolas, Forestales y Medio Ambiente, Centro de Investigación y Tecnología Agroalimentaria de Aragón (CITA), Zaragoza, Spain; 3 Instituto Agroalimentario de Aragón – IA2 (CITA-Universidad de Zaragoza), Zaragoza, Spain; 4 Área de Investigación Agroambiental, Instituto Madrileño de Investigación y Desarrollo Rural, Agrario y Alimentario (IMIDRA), Madrid, Spain

**Keywords:** *Xanthomonas euvesicatoria* pv. *euvesicatoria*, bacterial spot, pepper, lytic phage cocktail, *Beograduvirus*, biocontrol, One-Health, SDG

## Abstract

Bacterial spot of pepper and tomato is one of the most challenging xanthomonad diseases, affecting widely cultivated horticultural crops. *Xanthomonas euvesicatoria* pv. *euvesicatoria* (Xee), the main causal agent of bacterial spot in pepper, causes significant yield losses worldwide and is increasingly being reported in Southern Europe, including Spain, where outbreaks of bacterial spot have mainly been detected in pepper. Current management strategies mainly rely on copper-based compounds with limited efficacy due to the development of resistance and environmental and health concerns. Bacteriophages represent a promising safe and sustainable ecological alternative. In this study, lytic phages infecting Xee were isolated from pepper plots affected by bacterial spot disease in two distant regions of Spain. Genomic and taxonomic analysis of five selected phages classified them as belonging to the genus *Beograduvirus* and confirmed their lytic nature and safety. Three phages were investigated further because of their ability to infect multiple strains of the pathogen while remaining highly specific. These phages exhibited myovirus morphology and remained stable across temperatures and pH conditions relevant to field application. *In vitro* co-culture of the target bacteria with the three phages, either individually or in combination, revealed that the three combined phages controlled pathogen growth significantly better than single-phage treatments. In pepper plants, foliar application of the phage cocktail by dipping or spraying markedly reduced disease symptoms after inoculation with Xee, with spraying giving the most consistent results regardless of the bacterial strain tested. This is the first European phage cocktail that can effectively control Xee in pepper plants, highlighting its potential as a practical tool for the integrated management of this disease.

## 1 Introduction

The *Xanthomonas* genus comprises Gram-negative, yellow-colored plant-associated bacteria, belonging to the *Lysobacteraceae* family (syn: *Xanthomonadaceae*) (Naushad et al., 2015). This genus includes a wide range of phytopathogens responsible for severe diseases in over 400 plant species, including important crops such as rice, beans, tomatoes, peppers, citrus and fruit trees (Timilsina et al., 2020; Dey and Raghuwanshi, 2024). Infection starts with an epiphytic stage, where bacteria enter through wounds and natural openings into the aerial host tissues, and follows with an endophytic colonizing stage (An et al., 2020). One of the most challenging xanthomonad diseases is bacterial spot of pepper and tomato, where these pathogens cause damage to the leaves, stems and fruits, resulting in a loss of production and quality in both greenhouses and the field, particularly in hot and humid conditions (Horvath et al., 2012). Initial symptoms are water-soaked lesions on the aerial parts, which progress into chlorotic halos and necrotic spots (European and Mediterranean Plant Protection Organization [EPPO], 2023). Pepper (*Capsicum annuum* L.) is, along with potatoes and tomatoes, one of the most widely cultivated horticultural crops (Lozada et al., 2022; Hernández-Huerta et al., 2023). However, *X. euvesicatoria* may reduce the production yield in up to 66%, with losses of up to $7,500 US dollars per hectare (Jones et al., 2004; Sharma and Bhattarai, 2019; Hernández-Huerta et al., 2023), leading to significant economic losses worldwide (Potnis et al., 2015; Osdaghi et al., 2021; Giovanardi et al., 2025).

Currently, the etiological agents of bacterial spot of pepper and tomato are classified into four lineages within three *Xanthomonas* species: *X. euvesicatoria* pv. *euvesicatoria, X. euvesicatoria* pv. *perforans, X. hortorum* pv. *gardneri* and *X. vesicatoria* (Jones et al., 2004; Morinière et al., 2020; Osdaghi et al., 2021; European and Mediterranean Plant Protection Organization [EPPO], 2023). These phytopathogenic species are widespread and considered Regulated Non-Quarantine Pests (RNQP) in the European Union (EU) (European Commission, 2019). This means that, although present in the EU, specific measures are in place to limit their impact and prevent them from spreading further. In Spain, this disease has been found in pepper- and tomato-producing regions (López et al., 1985; Cazorla et al., 2000; Palacio-Bielsa et al., 2023), where outbreaks of bacterial spot were mainly detected in pepper, and to a lesser extent in tomato. The first Spanish isolates either from tomato or pepper were identified as *X. vesicatoria* (López et al., 1985; Cazorla et al., 2000). However, recent genomic characterization of Spanish strains responsible for outbreaks of bacterial spot in geographically distant regions has revealed that most isolates from pepper were *X. euvesicatoria* pv. *euvesicatoria*, whereas *X. vesicatoria* was mostly found in tomato (Palacio-Bielsa et al., 2023; Camañes et al., 2025). Therefore, in Spain, *X*. *euvesicatoria* pv. *euvesicatoria* affects peppers more severely than tomatoes.

Strategies for managing bacterial spot in pepper and tomato should include using pathogen-free seeds or seedlings, eliminating possible sources of inoculum, maintaining optimal temperature and water regimes, and growing varieties that are less susceptible to the disease (Giovanardi et al., 2025). The most common disease control strategies rely on copper-based products, either on their own or, in some countries outside the EU, in combination with antibiotics. However, they are not always effective, as these causal agents can survive in affected fields and spread easily under favorable conditions (Osdaghi et al., 2021). Moreover, chemical control poses a severe threat to global health, since it contributes to the development of resistance to copper and/or antibiotics (Lamichhane et al., 2018; Nakayinga et al., 2021; Giovanardi et al., 2025), and exacerbates the global problem of antimicrobial resistance in humans, animals, plants, and the environment (Mann et al., 2021; García et al., 2023; Greer et al., 2024). In addition, copper can accumulate in soil and surface water, resulting in phytotoxicity and a reduction in microbial biodiversity, impacting soil health and function (Alengebawy et al., 2021). The former, together with the growing restriction of agrochemicals in the European ecological policies and the increasing social demand for safer treatments for human health and the environment, has led to renewed interest in biologically-based control methods (Álvarez and Biosca, 2025).

Lytic bacteriophages (phages) that are specific to plant pathogenic bacteria without affecting the surrounding microbiota, could be an effective and safe way of protecting plants from bacterial diseases, due to their host specificity and bactericidal activity, and their ability to spread without affecting the beneficial bacteria when the target is present (Buttimer et al., 2017; Álvarez et al., 2019; Holtappels et al., 2021; Biosca et al., 2024; Álvarez and Biosca, 2025). Further, the use of phages as biocontrol agents contributes to achieving the United Nations’ Sustainable Development Goals (UN SDGs) and the European “One Health” strategy, as they form part of a natural, safe and sustainable solution (Álvarez and Biosca, 2025).

In recent years, the use of lytic bacteriophages, either individually or combined in cocktails, against economically important *Xanthomonas* species such as *X. arboricola*, *X. campestris, X. citri* or *X. oryzae* (Stefani et al., 2021) has been consistently described, highlighting the interest in this natural biocontrol approach. In fact, phages with potential against *Xanthomonas* spp. have been recently reviewed (Nakayinga et al., 2021; Stefani et al., 2021; Choudhary et al., 2025), including those active against *X. euvesicatoria* and *X. vesicatoria*. Furthermore, a *Xanthomonas* phage-based cocktail has been commercialized for the biological control of the bacterial spot disease caused by *X. euvesicatoria* pv. *perforans* in the USA (Choudhary et al., 2025). However, the limited availability of effective *X. euvesicatoria* pv. *euvesicatoria* specific phages in pepper plants restricts the development of cocktails addressed to control this pathogen.

To date, very few *X. euvesicatoria* pv. *euvesicatoria* phages with biological control capacity in plants have been described. Gašić et al. (2011) isolated the Serbian phage Kφ1 from the rhizosphere of pepper plants and showed its effectiveness in controlling bacterial spot when applied to pepper leaves under greenhouse conditions (Gašić et al., 2018). On the other hand, Kizheva et al. (2023) isolated and characterized 11 phages from the rhizosphere of tomato plants exhibiting bacterial spot symptoms in Bulgaria. They selected a single phage, BsXeu269p/3, and found that after applying it by spraying or injection to affected pepper leaves, the spread of bacterial spot under laboratory conditions could be reduced (Shopova et al., 2023). Nevertheless, in general, the use of individual phages as biological control agents carries the risk of the appearance of bacterial resistances, which is a major limitation for the development of effective treatments by phage-based methods. Moreover, the efficacy of phages as biocontrol agents also depends on the susceptibility of the target bacterium, as well as on environmental factors affecting bacteriophage survival. Thus, it is necessary to search for new bacteriophages that are effective and adapted to the environmental conditions of the affected areas.

This study aimed to develop an eco-sustainable and safe biological control strategy for the bacterial spot disease caused by *X. euvesicatoria* pv. *euvesicatoria*, based on the use of lytic phages in Mediterranean bioclimatic conditions. To this end, a collection of *X. euvesicatoria* pv. *euvesicatoria* phages was isolated from pepper plant plots affected by the disease in two geographically distant regions of Spain. Five phages were selected for genomic and phylogenetic characterization, being identified as members of the *Beograduvirus* genus. Their lytic nature and safety were also confirmed. *In vitro* biological characterization was used to further select three of the lytic and specific phages, which were assayed either alone or in combination. Subsequently, the effectiveness of a cocktail of the three selected phages in reducing bacterial spot symptoms was demonstrated when applied by dipping or foliar spraying to pepper plants inoculated with two different strains of the pathogen. This was found to be significantly effective regardless of the bacterial strain or application method. Thus, the development of a combination of European lytic and specific bacteriophages effective for controlling bacterial spot caused by *X. euvesicatoria* pv. *euvesicatoria* in pepper plants is firstly reported in this work.

## 2 Materials and methods

### 2.1 Bacterial strains and culture conditions

Spanish strains LSV 302 and CITA 26 of *X. euvesicatoria* pv. *euvesicatoria* (Xee) (Table 1), were used in all assays. Additional Xee strains isolated from different Spanish regions and years, as well as strains of *Xanthomonas* related species (Table 1), were used for bacteriophage host range and specificity assays. Further, strains of other bacterial species from the pepper plant microbiota and from other environments were used in phage assays as non-target bacteria (Supplementary Table 1). Strains were routinely grown in the general medium Luria Bertani (LB) broth (tryptone 10 g/L, yeast extract 5 g/L, NaCl 10 g/L) and on Nutrient Broth (NB) (peptone 5 g/L, beef extract 3 g/L, NaCl 5 g/L) alone or supplemented with agar (agar 15 g/L) (NA) at 28°C for 24 and 48 h, respectively. For phage isolation and amplification, LB was used at 28°C with shaking at 150 rpm. Strains were handled under biosafety levels 1 and 2 conditions in accordance with their biological risk level and the volume of inoculum used in certain experiments. All bacterial strains were cryopreserved in NB supplemented with 20 and 25% (v/v) glycerol at both -20°C and -80°C, respectively.

**TABLE 1:** Strains of *Xanthomonas euvesicatoria* pv. *euvesicatoria* and other *Xanthomonas* spp. pathogenic for tomato and pepper used in this study.

Bacterial species and strain	Host plant	Geographical origin	Isolation year
X. euvesicatoria pv. euvesicatoria			
Reference strains			
CFBP^a^ 6864^T^	*Capsicum frutescens*	USA	1947
LMG^b^ 930 (NCPPB^c^ 2574)	*C. frutescens*	USA	1969
Spanish strains			
IVIA^d^985-A1	*Capsicum annuum*	Murcia	1989
IVIA 985-A3	*C. annuum*	Murcia	1989
IVIA 1096-5	*C. annuum*	Comunidad Valenciana	1990
IVIA AP-Col 1	*C. annuum*	Almería	1991
IVIA AP-Col 3	*C. annuum*	Almería	1991
IVIA 1779-2	*C. annuum*	Ciudad Real	1997
IVIA 1780-1	*C. annuum*	Cuenca	1997
IVIA 1786-8a	*C. annuum*	Castilla—La Mancha	1997
IVIA 2133-1	*C. annuum*	Aragón	1999
IVIA 3580	*C. annuum*	Ciudad Real	2009
IVIA 3581	*C. annuum*	Ciudad Real	2009
IVIA 3617	*C. annuum*	Castilla—La Mancha	2009
IVIA 3619	*C. annuum*	Ciudad Real	2009
CRD^e^ 03/392	*C. annuum*	Castilla y León	2003
CRD 03/473	*C. annuum*	Castilla y León	2003
CRD 04/Xv10B	*C. annuum*	Castilla y León	2003
LPSVA^f^ B663	*C. annuum*	Andalucía	2011
LPSVA B730	*C. annuum*	Andalucía	2014
LPSVA B746	*C. annuum*	Andalucía	2014
LPSVA B768	*Solanum lycopersicum*	Andalucía	2015
LPSVA B769	*C. annuum*	Andalucía	2015
LPSVA B770	*C. annuum*	Andalucía	2015
LPSVA B830	*C. annuum*	Andalucía	2017
LSV^g^302	*C. annuum*	Extremadura	2024
UV^h^52-9.1	*C. annuum*	Extremadura	2024
UV 52-11.1	*C. annuum*	Extremadura	2024
UV53-2.1	*C. annuum*	Extremadura	2024
UV53-5.1	*C. annuum*	Extremadura	2024
CITA^i^23	*C. annuum*	Aragón	2024
CITA 24	*C. annuum*	Aragón	2024
CITA 26	*C. annuum*	Aragón	2024
UV 54-2	*C. annuum*	Aragón	2024
UV 54-11	*C. annuum*	Aragón	2024
UV 54-24	*C. annuum*	Aragón	2024
X. euvesicatoria pv. perforans			
CFBP 7293**^T^**	*S. lycopersicum*	Florida	1991
CFBP 7993	*S. lycopersicum*	Mauricio island	2010
X. hortorum pv. gardneri			
CFBP 8163^T^	*S. lycopersicum*	Yugoslavia	1953
CFBP 7999	*S. lycopersicum*	New Zeland	1980
CFBP 8588	*S. lycopersicum*	Reunion island	1997
X. vesicatoria			
CFBP 2537**^T^**	Unknown	New Zealand	1955
CFBP 1941	*S. lycopersicum*	Murcia	1978
CECT^j^ 792	Unknown	Israel	Unknown

^a^CIRM-CFBP, International Centre of Microbial Resource—French Collection for Plant-associated Bacteria, Institute National de Recherche pour l’Agriculture, l’Alimentation et l’Environnement, France;

^b^LMG, Belgian Coordinated Collections of Microrganisms;

^c^NCPPB, National Collection of Plant Pathogenic Bacteria, York, United Kingdom;

^d^IVIA, Instituto Valenciano de Investigaciones Agrarias, Valencia, Spain;

^e^CDR, Centro Regional de Diagnóstico Junta de Castilla y León, Salamanca, Spain;

^f^LPSVA, Laboratorio de Producción y Sanidad Vegetal de Almería, Spain;

^g^LSV, Laboratorio de Diagnóstico de Sanidad Vegetal Junta de Extremadura, Badajoz, Spain;

^h^UV, Universitat de Valéncia, Valencia, Spain;

^i^CITA, Centro de Investigación y Tecnología Agroalimentaria de Aragón, Zaragoza, Spain;

^j^CECT, Colección Española de Cultivos Tipo, Valencia, Spain; T, Type strain.

### 2.2 Bacterial and phage isolation

In the summer of 2024, two pepper crop plots in Extremadura and one in Aragón (western and eastern regions of Spain, respectively), which had experienced localized outbreaks of pepper bacterial spot, were sampled. Leaves, fruits, stems, and roots from symptomatic pepper plants, as well as soil taken from the substrate beneath the affected plants, and irrigation water, were analyzed. Bacterial and phage isolation was performed concurrently to ensure a thorough evaluation. For phage isolation, the Xee strains LSV 302 and CITA 26 were used as host bacteria. These strains were previously isolated from the pepper-affected plots in Extremadura and Aragón, respectively, and identified by the respective Plant Health Laboratories, which supplied them for this study, as well as the pepper plot samples. Additionally, bacterial isolation was performed to find new local Xee strains for phage isolation and host range assays. Further, pepper plant-associated bacteria were isolated from the same samples for phage specificity analysis.

Bacterial and phage isolation was performed directly from samples of 5 g of each type of plant material (leaves, fruits, stems, and roots with associated rhizosphere) per plot. At least four different symptomatic plants were sampled at different points within each plot. The samples were then grounded in 20 mL of SM buffer (50 mM Tris-HCl, pH 7.5; 100 mM NaCl; 10 mM MgSO_4_; and 0.01% gelatine). The resulting crushed plant material was inoculated on NA plates for bacterial isolation. Some of these NA plates were used for the direct isolation of phages. This involved successively filtering the samples of plant extracts through sterile filters with pore diameters of 0.45 and 0.22 μm, and inoculating plates that had been pre-inoculated with the Xee LSV 302 or CITA 26 strains for samples from Extremadura and Aragón, respectively.

Phage isolation was also carried out by enrichment of filtered plant extracts by mixing them at a ratio of 1:1 with LB medium 2X that had been inoculated separately with either strain Xee LSV 302 or CITA 26, depending on the geographical origin of the samples. To increase the likelihood of isolating phages from the samples, enrichment was also performed using a mixture of strains, including one of those mentioned above, combined with the following Xee reference strains: CFBP 6864, CDR 03/392, CDR 03/473, and CDR 04/Xv10B. In addition, an enrichment was performed with the reference strain of *X. vesicatoria* CFBP 1941 to try to isolate phages that can lyse strains of this species closely related to Xee, which has also been responsible for bacterial spot outbreaks in Spain. Prior to inoculation, all Xee strains were adjusted to an optical density at 600 nm (OD_600_) of 0.5 and then incubated at 28°C with shaking at 150 rpm for 24 and 48 h. Similarly, samples of 20 g or 20 mL of soil beneath the infected plants or irrigation water, respectively, from 4 different sampling points per plot, were mixed with LB 2X and inoculated with Xee strains as described above.

All enrichments (plant material, water and soil) were centrifuged twice at 4,000 rpm for 10 min and the supernatants were sequentially filtered through 0.45 and 0.22 μm pore-size filters to obtain bacterium-free phage suspensions. During the enrichment process, LB medium inoculated with host bacteria alone and LB medium without bacterial inoculation were included as positive and negative controls, respectively.

### 2.3 Bacterial identification

A collection of presumptive Xee yellow colonies obtained from NA isolation plates inoculated with pepper plant extracts, were selected, purified, and identified by PCR following the protocol described by Koenraadt et al. (2009), according to European and Mediterranean Plant Protection Organization [EPPO] (2023), using the specific primers Bs-XeF (5′-CATGAAGAACTCGGCGTATCG-3′) and Bs-XeR (5′-GTCGGACATAGTGGACACATAC-3′). Xee strains LSV 302 and CITA 26 were used as positive controls, and strain CFBP 1941 of *X. vesicatoria* was used as a negative control, as well as water.

Additionally, diverse bacterial colonies with different morphology to *Xanthomonas* spp. were randomly selected from these same NA isolation plates. These pepper plant bacterial isolates were then purified and identified using 16S ribosomal RNA (*16S rRNA*) gene sequencing. Briefly, partial amplification of the *16S rRNA* gene from the bacterial isolates was performed by PCR using primers 616V (5′-AGAGTTTGATYMTGGCTCAG-3′) and 699R (5′-GGGTYKCGCTCGTTR-3′), following the protocol described by Arahal et al. (2008). After electrophoretic separation and band visualization, the resulting fragments were purified and sequenced by the Sanger method at the Sequencing Service of the Central Experimental Research Service (SCSIE) at the University of Valencia (Spain). The obtained partial *16S rRNA* gene sequences from plant-associated bacterial isolates were analyzed using BLASTN against the NCBI nucleotide database for taxonomic identification at the genus level, based on the highest sequence similarity and query coverage.

### 2.4 Pathogenicity of the *X. euvesicatoria* pv. *euvesicatoria* strains

The pathogenicity of the Xee strains LSV 302 and CITA 26 was confirmed on plants of the susceptible Spanish commercial pepper variety “Piquillo,” which were approximately 7–10 cm tall and had 7–8 fully expanded leaves. Different bacterial concentrations (10^6^, 10^7^, and 10^8^ colony-forming units (CFU)/mL) were inoculated by leaf dipping following Tamir-Ariel et al. (2007), with minor modifications, using six plants per concentration and strain. Briefly, leaves were immersed in the corresponding bacterial suspension to ensure uniform and complete contact with the leaf surface. This approach allowed for monitoring of symptoms and determination of the bacterial dose to be used in subsequent biocontrol assays. After inoculation, plants were enclosed in transparent plastic bags to maintain high relative humidity level, following European and Mediterranean Plant Protection Organization [EPPO] (2023) recommendations, and subsequently maintained in a controlled-environment growth chamber (Panasonic MLR-352-PE) at 26°C under a 16 h light/8 h dark photoperiod.

Once the lowest concentration of the bacterial pathogen inducing early and consistent symptoms was selected (10^7^ CFU/mL), the assay was repeated using this concentration to confirm reproducibility and to quantify disease severity by counting the number of lesions per plant.

Hypersensitive response (HR) assays were performed on tobacco (*Nicotiana benthamiana*) leaves using suspensions of strains Xee LSV 302 and CITA 26, including a positive control (CFBP 1554 strain of *Erwinia amylovora*, Supplementary Table 1) and a negative control (Phosphate-Buffered Saline (PBS) buffer, pH 7.2) similar to Santander et al. (2018). Briefly, bacterial suspensions at a concentration of 10^8^ CFU/mL were infiltrated into the veins of the leaves using a syringe, after which the infiltration area was labeled. This test was performed in duplicate. The development of necrotic lesions was evaluated after 72 h.

### 2.5 Selection and amplification of *X. euvesicatoria* pv. *euvesicatoria* phages

Plaque-forming units (PFUs) exhibiting different morphologies were initially selected according to various criteria, such as size and clarity, indicative of lytic activity, as well as well-defined edges. Single plaques were collected using a sterile pipette tip and incubated overnight in SM buffer. The suspensions were then centrifuged, and the resulting supernatant was filtered through 0.22 μm sterile filters and then serially 10-fold diluted. Each dilution was co-inoculated with the Xee host strain using the double-layer agar method and incubated at 28°C for 24–48 h, similarly to Biosca et al. (2024). This process was repeated until purified phages were obtained. The stability of plaque morphology during purification was an additional criterion for phage selection.

After purification, phages were propagated in LB medium with a fresh culture of the host strains and titrated as PFU/mL using the agar double-layer assay. Bacterial positive controls (LB with host bacteria) and negative controls (LB without bacteria nor phages) were included. After 24 h of incubation, cultures showing lysis were centrifuged (10,000 rpm, 10 min) and the supernatants were filtered through sterile filters with a pore size of 0.22 μm. Short-term storage of purified phage suspensions was carried out at 4°C in LB broth, while long-term storage involved cryopreservation at -20°C and -80°C in LB broth supplemented with 25 and 30% glycerol (v/v), respectively. Additional criteria for phage selection included phage amplification rates, geographical origin, pepper plot, and source of isolation (plant material, soil or irrigation water).

### 2.6 Genomic characterization and comparative analysis of the five *X. euvesicatoria* pv. *euvesicatoria* phages

Phages P4A, P8B, P10B, P4A, and W18B, representing different sampling sources and locations (Table 2), were selected for whole-genome sequencing based on the criteria described above. In phage designation, “P” refers to plant material and “W” to irrigation water. The letter “A” indicates isolation using a mixture of host strains, whereas “B” denotes isolation using a single host strain. Bacterial nucleic acids in filtered phage lysates were removed by DNase and RNase treatment for 1 h at 37°C, followed by enzyme inactivation with EDTA at 37°C for 10 min. Phage DNA was extracted using the CTAB protocol (Murray and Thompson, 1980). DNA concentration, purity and integrity were evaluated using a NanoDrop spectrophotometer, and 1% agarose gel electrophoresis.

**TABLE 2:** Geographical origin, genomic features and host range of *Xanthomonas euvesicatoria* pv *euvesicatoria* (Xee) phages from pepper plants.

Phage	Plot/Spanish region	Source	Genome size (bp)	GC (%)	GenBank accession	Complete lysis^a^ (%)
vXeeB-P4A	P1/Extremadura	Root and rhizosphere-associated	47,892	62.94%	PZ019799	88
vXeeB-P8B	P2/Extremadura	Fruits	47,424	62.91%	PZ019800	88
vXeeB-P10B	P3/Aragón	Root and rhizosphere-associated	47,421	62.92%	PZ019801	91
vXeeB-P14A	P3/Aragón	Fruits	47,410	62.92%	PZ019802	83
vXeeB-W18B	P3/Aragón	Water	47,424	62.94%	PZ019803	83

^a^Percentage of Xee strains on which phages have shown complete lysis. See Figure 5 for further information.

Sequencing was performed on a MinION Mk1B (Oxford Nanopore Technologies, Oxford, United Kingdom) using the Rapid Barcoding Kit 24 and an R10.4.1 flow cell. Basecalling was carried out with Dorado v7.4.14 (Super-accurate base-calling v4.3.0, 400 bps).^1^ Raw reads were processed with the nf-core/bacass v2.4.0 pipeline (Vm et al., 2024), which includes read trimming, quality assessment, contamination screening, and *de novo* assembly with Unicycler (Wick et al., 2017). Contigs were analyzed by BLASTn and BLASTx, and completed genomes were annotated using Pharokka v1.7.5 (Bouras et al., 2023). The prediction of lytic or lysogenic lifecycle, as well as the search for antimicrobial resistance and virulence factors, was performed using PhageScope (Wang et al., 2024). All assembled genomes were deposited to the NCBI GenBank database.

To infer the DNA packaging strategy of the phages characterized in this study, a phylogenetic analysis of the large terminase subunit (*terL*) was performed following the approach described by Biosca et al. (2021). Briefly, a dataset comprising 50 amino acid reference sequences, representative of the main DNA packaging strategies described in tailed bacteriophages, were aligned together phages studied using Clustal Omega and phylogenetic reconstruction was carried out with FastTree v2.1.11 under default parameters, as implemented in Geneious Prime (2026). To further characterize genome termini and DNA packaging mechanisms, high-coverage (2 × 250 bp) paired-end reads generated on an Illumina MiSeq platform using the Illumina DNA Prep kit were analyzed with PhageTerm for phages vXeeB-P4A and vXeeB-P8B (Garneau et al., 2017).

For comparative genomic analyses, the phage genomes were compared with all available members of the genus *Beograduvirus* (Subfamily*: Kantovirinae*) constituted for the *Xanthomonas* phages: *Xanthomonas* phage phiXaf18 (NC_054461.1), *Xanthomonas* phage MYK3 (OK275494.1), *Xanthomonas* phage Kφ1 (NC_054460.1), and *Xanthomonas* phage BsXeu269p/3 (ON996340.1). Pairwise intergenomic similarities were calculated using VIRIDIC (Moraru et al., 2020), while Average Nucleotide Identity (ANI) and Alignment Percentage (AP) were determined with QIAGEN CLC Genomics Workbench v25.0.3. Locus visualizations were generated using LoVis4u v0.1.6 (Egorov and Atkinson, 2025).

### 2.7 Host range and specificity of *X. euvesicatoria* pv. *euvesicatoria* phages

The host range and specificity of the five selected phages were assessed using a spot test adapted from that described by Biosca et al. (2024). Spanish strains of Xee and other *Xanthomonas* species causing bacterial spot disease, including reference strains (Table 1), as well as bacterial isolates from pepper plant–associated microbiota and strains from other environments (Supplementary Table 2), were used in these assays. Bacterial lawns were prepared on NA soft agar plates.

A 10 μL drop of each phage suspension (10^8^ PFU/mL) was spotted in duplicate onto the surface of the NA top agar inoculated with each bacterial strain. Plates were incubated at 28°C overnight. Strains LSV 302 and CITA 26 were included as controls for positive phage sensitivity in all assays. The presence of clear or turbid lysis zones at the site of drop deposition was recorded as indicative of phage sensitivity, whereas the absence of visible changes was considered negative. In addition, lytic activity against Xee strains was also evaluated in liquid LB medium. All assays were performed in at least two independent experiments.

### 2.8 Morphological characterization of three selected *X. euvesicatoria* pv. *euvesicatoria* phages

Suspensions of virions of three selected phages were prepared according to the method described by Biosca et al. (2024). Five microliters of these suspensions were deposited on copper grids coated with formvar and carbon, and the excess was removed. The phages were then stained with 1% phosphotungstic acid (pH 7.0), and were then visualized using a Hitachi HT7800 transmission electron microscope (100 kV) equipped with a 20 MPx EMSIS XAROSA digital camera, controlled by RADIUS software at the facilities of the Central Experimental Research Support Service (SCSIE, University of València, Spain). Image J software (version 1.53 m) (Rasband, 2022) was used to determine the dimensions of the virions (capsid diameter and tail length) in micrographs, with measurements taken from at least 10 capsids and 10 tails per phage.

### 2.9 Survival of the three phages at different temperatures and pH values

*In vitro* survival assays were performed for the three selected phages from different plots and geographical origins (P4A, P8B, and P10B) at three temperatures and three pH conditions over the course of one month. The phage suspensions were adjusted to a final concentration of 10^8^ PFU/mL in SM buffer. To study the influence of temperature on phage stability, the suspensions were incubated at 4, 28, and 37°C in SM buffer at pH 7.2. The effect of pH on phage survival was investigated by adjusting the SM buffer to pH 5.5, 7.2, or 8.0, preparing the phage suspensions at these values and incubating them at 28°C. Phage survival was estimated by determining the titers by counting the PFU/mL of suspensions taken at 0, 1, 2, 3, and 4 weeks of incubation using the double-layer agar method after 24 h of incubation of the plates at 28°C. All assays were performed in duplicate, with triplicate plaque counts per assay, and the results were expressed as the mean PFU/mL ± standard deviation.

### 2.10 Biocontrol of *X. euvesicatoria* pv. *euvesicatoria* by the three selected phages

#### 2.10.1 Biocontrol in co-culture using three single phages and their combination into a cocktail

The dynamics of the lytic infection of Xee by phages was evaluated *in vitro* by monitoring their co-culture with host strains, as described by Biosca et al. (2024), with minor modifications. Bacterial suspensions of strains LSV 302 and CITA 26 were adjusted spectrophotometrically to 10^8^ CFU/mL (OD_600_ = 0.1) and subsequently tenfold diluted to obtain a final concentration of 10^7^ CFU/mL. Phage suspensions were then added at a final concentration of 10^8^ PFU/mL, corresponding to a multiplicity of infection (MOI) of 10, to reproduce the bacteria-to-phage ratio used in the *in planta* experiments. Each phage was evaluated individually against its corresponding host strain of isolation. In addition, all three phages were combined into a single cocktail and evaluated at a MOI of 10 using Xee strain CITA 26 at 10^7^ CFU/mL. Each phage was also individually tested against this strain under the same conditions, regardless of the isolation host.

Phage–bacteria interactions were monitored in microtiter plates by recording OD_600_ every 120 min for 48 h at 28°C with shaking, using a spectrophotometer plate reader (Tecan Infinite M Nano, Männedorf, Switzerland). LB medium with and without bacterial inoculation served as controls. At least three technical replicates per condition were performed in two independent experiments.

#### 2.10.2 Biocontrol *in planta* with the cocktail of the three phages

The biocontrol efficacy of a three-phage cocktail against Xee was assessed in terms of its ability to prevent or reduce bacterial spot symptoms in pepper plants of variety “Piquillo,” which were approximately 7–10 cm tall and had 7–8 fully expanded leaves.

Biocontrol assays were conducted using strains LSV 302 and CITA 26, at a MOI of 10 (bacteria at 10^7^ CFU/mL:phages at 10^8^ PFU/mL). Positive controls consisting of plants inoculated only with the bacterial strains (LSV 302 or CITA 26), as well as two negative controls comprising PBS and the phage cocktail alone in SM buffer, were included.

The efficacy of the phage cocktail was evaluated using two application methods: leaf dipping (as described above, in the pathogenicity assays; Tamir-Ariel et al., 2007) and foliar spraying (Shopova et al., 2023). In the spraying assay, bacterial, phage or bacterium–phage suspensions, as well as PBS, were uniformly applied to the leaf surface using a handheld sprayer to ensure homogeneous coverage. In both methods, plants were visually inspected to confirm that all leaves were evenly inoculated. Inoculated plants were placed in transparent plastic bags to maintain a high level of relative humidity and were incubated in a Panasonic MLR-352-PE controlled-environment growth chamber at 26°C under a photoperiod of 16 h light and 8 h darkness for 3 weeks. At least three independent experiments were conducted, each of them comprising groups of six pepper plants per condition, application method and bacterial strain. The efficacy of the phage cocktail was determined by counting the number of lesions on the inoculated leaves at the end of the experiment and normalizing the values by the leaf surface area per plant, measured using ImageJ software (version 1.54p; Schindelin et al., 2012).

To verify the presence of Xee in symptomatic plants, bacteriological analyses were conducted on a subset of plants exhibiting characteristic disease lesions. Symptomatic leaf tissues were processed, and presumptive yellow colonies were isolated on NA plates as described above. The identification of random selected and purified isolates was conducted by PCR, utilizing the species-specific primers Bs-XeF and Bs-XeR (Koenraadt et al., 2009).

### 2.11 Statistical analysis

All statistical analyses were conducted in R (v4.4.2). Model assumptions (normality and homogeneity of variances) were assessed through residual diagnostics, Shapiro–Wilk and Levene’s tests when appropriate. When significant effects were detected, pairwise comparisons were performed using estimated marginal means with Tukey or Bonferroni adjustment (*p* < 0.05). Phage survival under different pH and temperature conditions was analyzed using linear mixed-effects models. Log-transformed titers were modeled as a function of time, treatment, phage, and their interactions, with replicate included as a random effect. Decay rates (slopes) were derived from model estimates. For *in vitro* biocontrol assays, bacterial growth was quantified as the area under the curve (AUC) calculated from OD_600_ measurements over time, as previously described by Biosca et al. (2024). AUC values were analyzed using one-way ANOVA. Disease severity data (lesions per leaf area) were log-transformed [log (x + 0.01)] and analyzed using a three-way ANOVA (Type III sums of squares) to evaluate the effects of treatment, inoculation method, bacterial strain, and their interactions.

## 3 Results and discussion

### 3.1 *X. euvesicatoria* pv. *euvesicatoria* identification and pathogenicity

**FIGURE 1:**
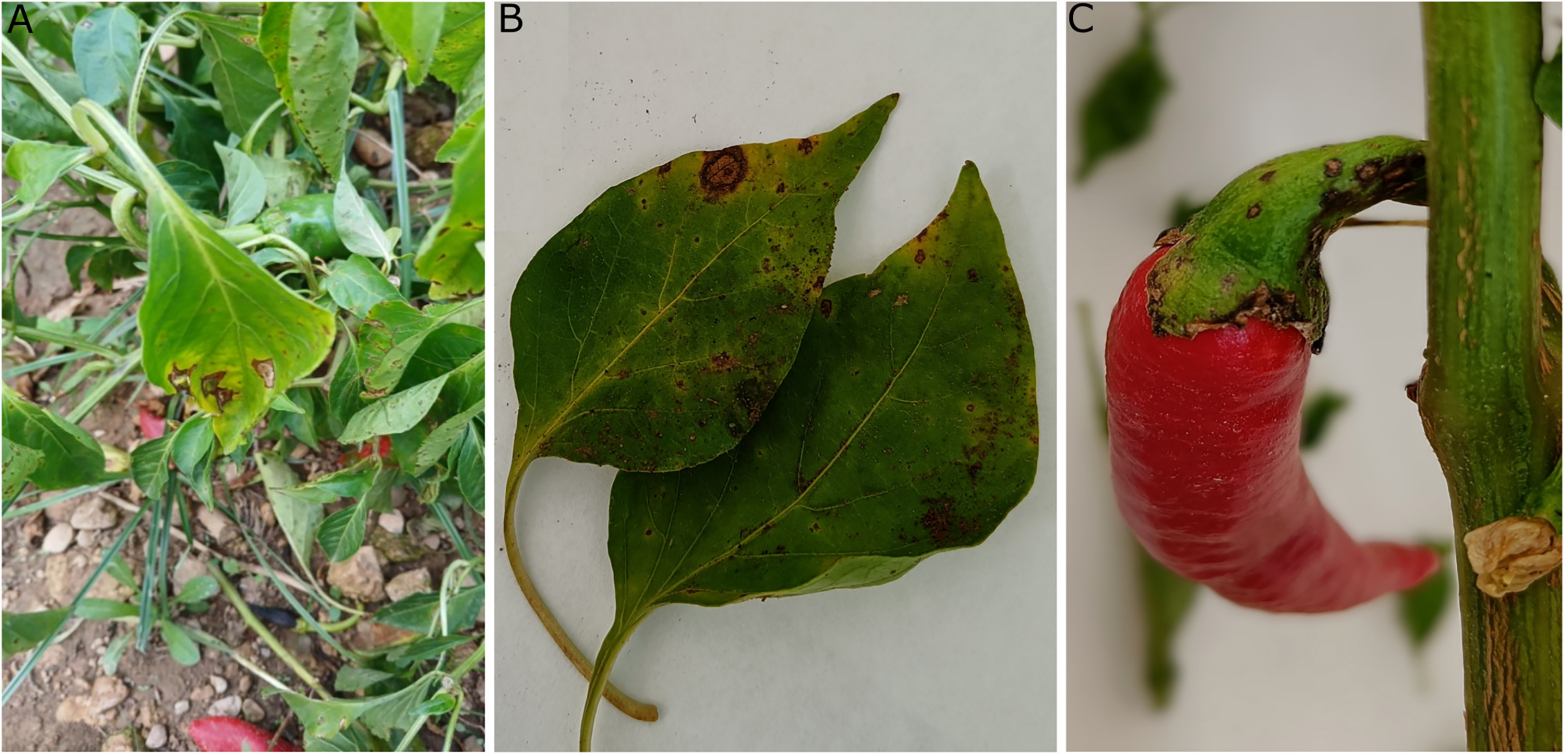
Symptoms of bacterial spot caused by *Xanthomonas euvesicatoria* pv. *euvesicatoria* on pepper plants in plots from Extremadura and Aragón **(A)** *in situ*, **(B)** on leaves, and **(C)** on pepper fruits.

**FIGURE 2:**
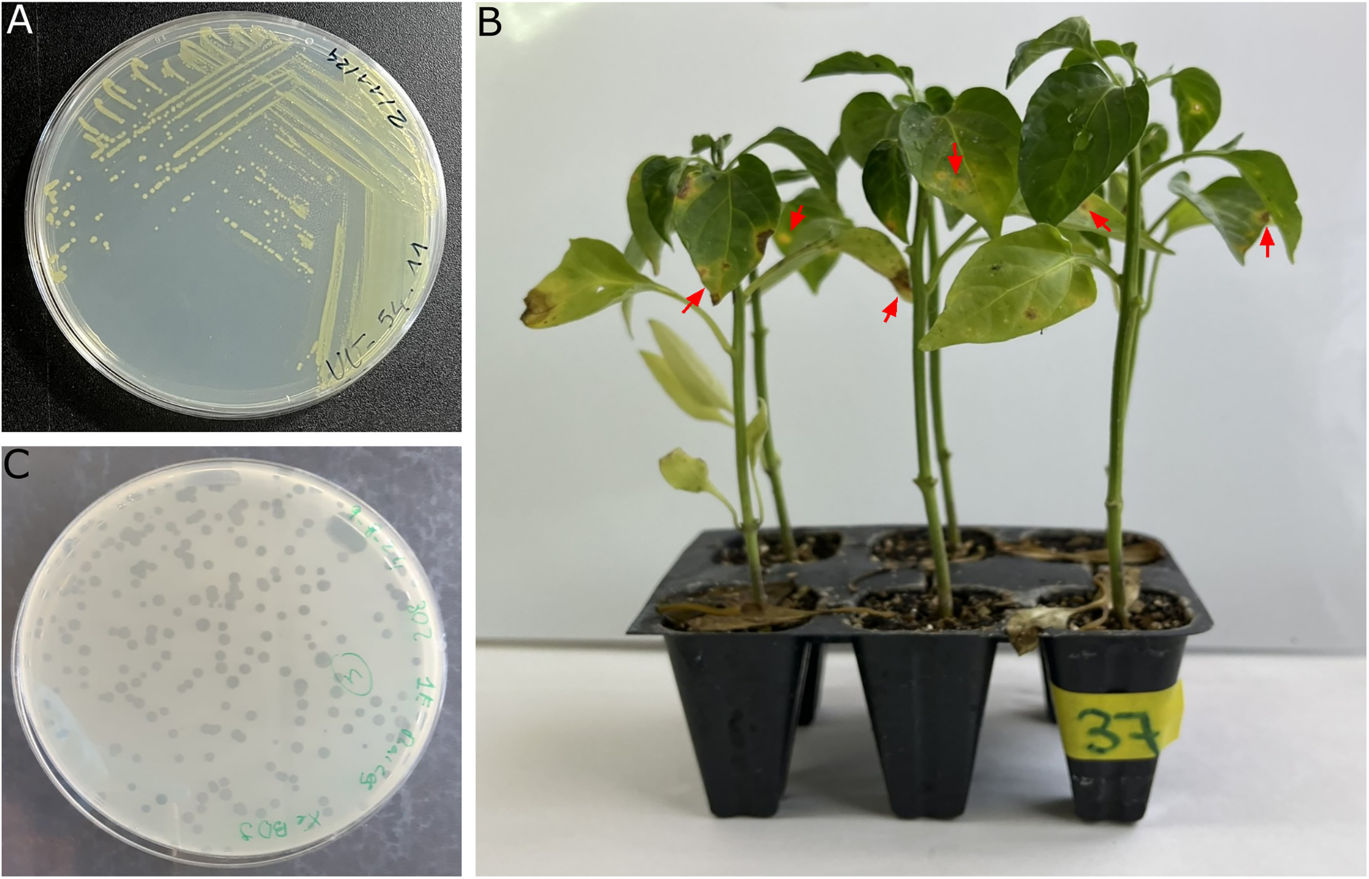
Isolation and pathogenicity assessment of *Xanthomonas euvesicatoria* pv. *euvesicatoria* (Xee), and isolated phages. **(A)** Three-strip plate purification of a selected bacterial isolate on NA prior to molecular confirmation as Xee. **(B)** Pathogenicity assay on pepper plants 21 days post-inoculation with Xee strain CITA 26, showing characteristic bacterial spot symptoms; red arrows indicate typical lesions. **(C)** Representative lysis plaques with distinct morphologies recovered from symptomatic plant material during phage isolation, as observed on NA plates inoculated with the Xee host strain after 48 h of incubation.

The fruits, leaves, roots and stems of pepper plants exhibiting symptoms of bacterial spot disease in two plots in Extremadura and one plot in Aragón (Figures 1A–C) were analyzed. As a result, yellow bacterial colonies exhibiting the characteristic morphology of *Xanthomonas* were obtained on NA (Figure 2A). After purification, seven isolates per plot were randomly selected and analyzed by PCR using Bs-XeF/Bs-XeR primers. Confirmation of Xee identity was obtained for 73% of the isolates through amplification of the specific 173 bp fragment. Representative confirmed isolates were selected for further characterization. Strains LSV 302 and CITA 26 were also tested by PCR and proved to be positive for Xee.

The pathogenicity of Xee strains LSV 302 and CITA 26 was evaluated on pepper plants using inoculum concentrations of 10^6^, 10^7^, and 10^8^ CFU/mL. Typical bacterial spot lesions appeared between 15 and 18 days post-inoculation (dpi) in plants inoculated with 10^7^ and 10^8^ CFU/mL, respectively, reaching full symptom development by 21 dpi (Figure 2B). By contrast, a concentration of 10^6^ CFU/mL resulted in delayed and milder symptoms. No differences were observed between 10^7^ and 10^8^ CFU/mL in terms of symptom onset or disease progression within the experimental period.

Based on these results, 10^7^ CFU/mL—the lowest concentration inducing early and consistent symptoms—was selected for performing subsequent experiments of this kind, in agreement with the European and Mediterranean Plant Protection Organization [EPPO] (2023) guidelines for pathogenicity assays. At 21 dpi, disease severity averaged 13.7 ± 7.7 lesions per plant for LSV 302 and 10.3 ± 3.9 for CITA 26. Given their clear and reproducible pathogenicity, both strains were used in subsequent assays, including *in planta* biocontrol trials, to account for potential strain-dependent effects.

The ability of Xee strains to induce the HR in non-host plants was also evaluated. As a result, this response was observed on tobacco plants inoculated with these two strains and also with newly isolated Xee strains (UV 52-11.1, UV53-2.1, UV53-5.1, UV 54-2, UV 54-11, UV 54-24) after 3 days of incubation, similar to the response of the strain of *E. amylovora* used as positive control. No HR was observed when plants were inoculated with sterile PBS.

### 3.2 Identification of pepper plant associated bacteria

Several morphologically distinct bacterial colonies clearly different from *Xanthomonas* spp. were isolated from pepper plant extracts, from plants collected in plots affected by bacterial spot. Ten of these isolates were purified and subjected to partial *16S rRNA* gene sequencing (Arahal et al., 2008). The obtained sequences showed similarities ranging from 98 to 99% with GenBank entries and were therefore assigned to the genera *Arthrobacter* (*n* = 1), *Microbacterium* (*n* = 5), *Pseudoclavibacter* (*n* = 1), and *Pseudomonas* (*n* = 3) (Supplementary Table 2). Most of the identified genera corresponds to bacterial taxa that have previously been described as endophytes of healthy pepper plants (Rasche et al., 2006; Chandra Paul et al., 2013). However, members of *Microbacterium*, *Pseudomonas*, and *Pseudoclavibacter* have also been associated with spoilage of fruits and vegetables, including peppers (Ballan et al., 2025), highlighting the ecological diversity and functional versatility of the pepper-associated microbiota.

### 3.3 *X. euvesicatoria* pv. *euvesicatoria*- specific phages were primarily isolated from infected pepper plants

Phages infecting Xee strains were detected in plant material and irrigation water taken from pepper plots where the pathogen was present. Phage isolation was performed both directly and following an enrichment step using selected Xee strains as hosts, alone or combined, as well as one strain of the close pathogenic species *X. vesicatoria*. After incubation, transparent and turbid plaques were observed on double-layer agar plates from plant extracts, soil suspensions, and irrigation water. An initial collection of 37 phages was obtained from the resulting transparent round lytic plaques (Figure 2C), ranging from 1 to 3 mm in diameter, across the three outbreak-affected plots. Most of the selected phage isolates (94.6%, *n* = 35) were recovered from diseased plant tissues, whereas the remaining selected isolates (5.4%, *n* = 2) were obtained from irrigation water samples. Plant-derived phages were predominantly recovered from root and rhizosphere-associated samples (49%, *n* = 17), followed by symptomatic leaves (34%, *n* = 12) and fruits (17%, *n* = 6).

These results are consistent with previous studies describing symptomatic plant tissues and the rhizosphere of diseased plants as common reservoirs of Xee-infecting phages (Gašić et al., 2011; Kizheva et al., 2023), with wastewater also reported as an additional source (Domingo-Calap et al., 2022). By contrast, irrigation water has rarely been reported as a source of Xee phages. No phages were isolated when the Spanish reference strain *X. vesicatoria* CFBP 1941 was used as the host.

Five phages (P4A, P8B, P10B, P14B, and W18B) were selected for genomic characterization (Table 2) based on plaque stability, high amplification capacity (reaching titers of up to 10^9^ PFU/mL after overnight propagation), geographical origin, sampling plot, and source of isolation (plant material or irrigation water).

### 3.4 *X. euvesicatoria* pv. *euvesicatoria* phages belong to *Beograduvirus*

Complete genome sequences were obtained for the five selected Xee phages (P4A, P8B, P10B, P14 B, and W18B) with genome sizes ranging from 47,410 to 47,892 bp and a uniform GC content of 62.9% (Table 2). Genome annotation with Pharokka predicted 76–79 coding sequences (CDS) per genome. No genes associated with antimicrobial resistance or virulence were detected, and all phages were predicted to exhibit a lytic lifecycle according to PhageScope. The complete genome sequences have been deposited in the GenBank database under accession numbers PZ019799- PZ019803.

**FIGURE 3:**
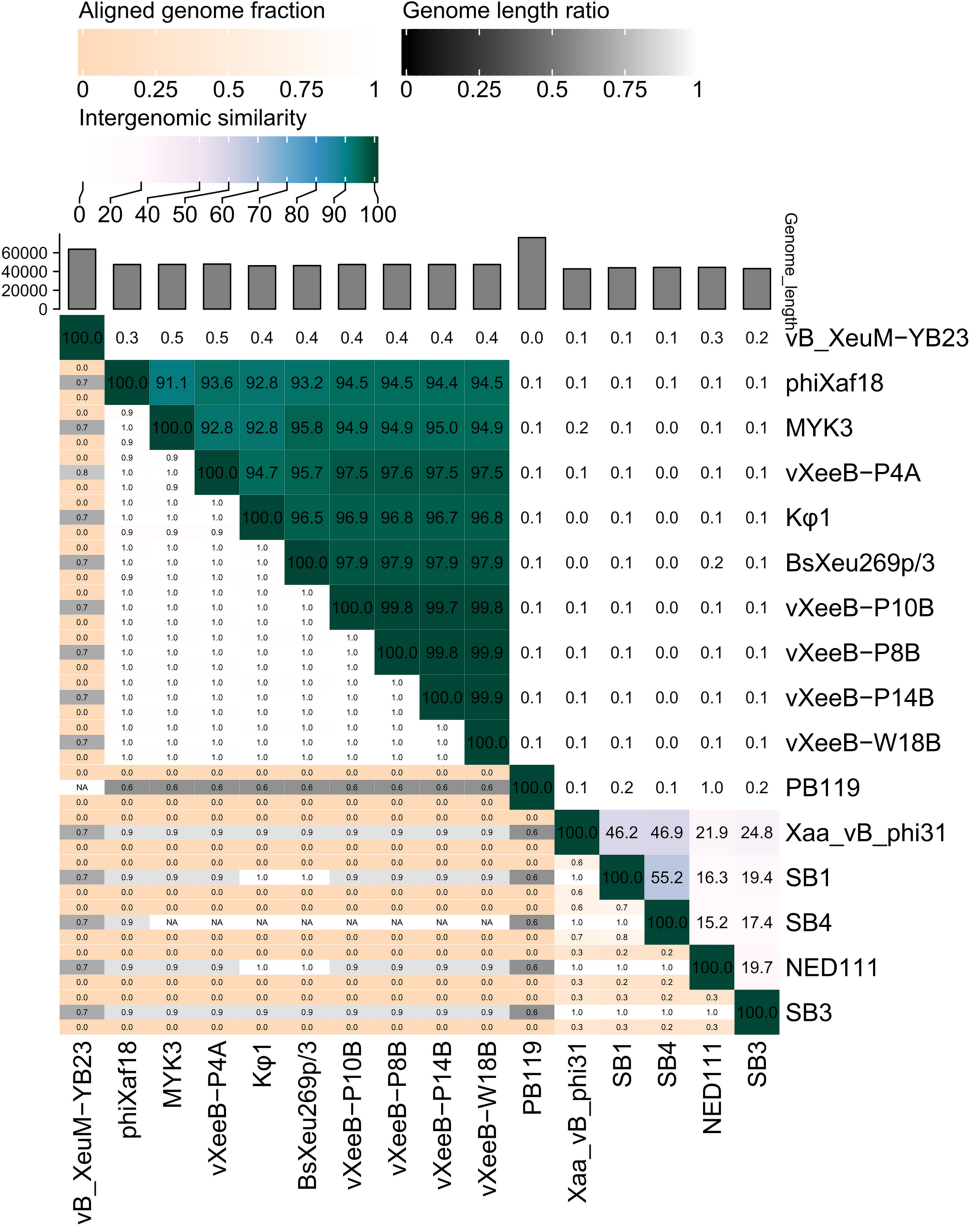
Heatmap showing pairwise intergenomic similarities among *Xanthomonas euvesicatoria* pv. *euvesicatoria* (Xee) phages (vXeeB-P4A, vXeeB-P8B, vXeeB-P10B, vXeeB-P14B, and vXeeB-W18B) and all available members of the genus *Beograduvirus* and related genera. The analysis was performed with VIRIDIC using complete genome sequences.

Comparative genomic analysis using VIRIDIC (Figure 3) revealed high intergenomic similarity (97.7–99.9%) among the five Xee phages, indicating they belong to the same species of the genus *Beograduvirus*. Then, they were named as vXeeB-P4A, vXeeB-P8B, vXeeB-P10B, vXeeB-P14B, and vXeeB-W18B. When compared with reference members of the genus *Beograduvirus*, the Xee phages vXeeB-P8B, vXeeB-P10B, vXeeB-P14B, and vXeeB-W18B showed 96.7–97.9% similarity to *Xanthomonas* phages Kφ1, which was obtained from the rhizosphere of diseased pepper plants in Serbia (Gašić et al., 2018), and BsXeu269p/3, which was isolated in Bulgaria from the rhizosphere of tomato plants affected by bacterial spot disease in a garden (Shopova et al., 2023), whereas the Xee phage vXeeB-P4A showed slightly lower identity values (94.7–95.7%). According to ICTV proposal 2021.038B, species demarcation within the subfamily *Kantovirinae* is defined by a threshold of ≥ 95% nucleotide identity across the complete genome. Based on these results, vXeeB-P8B, vXeeB-P10B, vXeeB-P14B, and vXeeB-W18B belong to the same species as *Beograduvirus* Kφ1, whereas vXeeB-P4A is closely related to the Kφ1 species, but showing slight genetic divergence.

**FIGURE 4:**
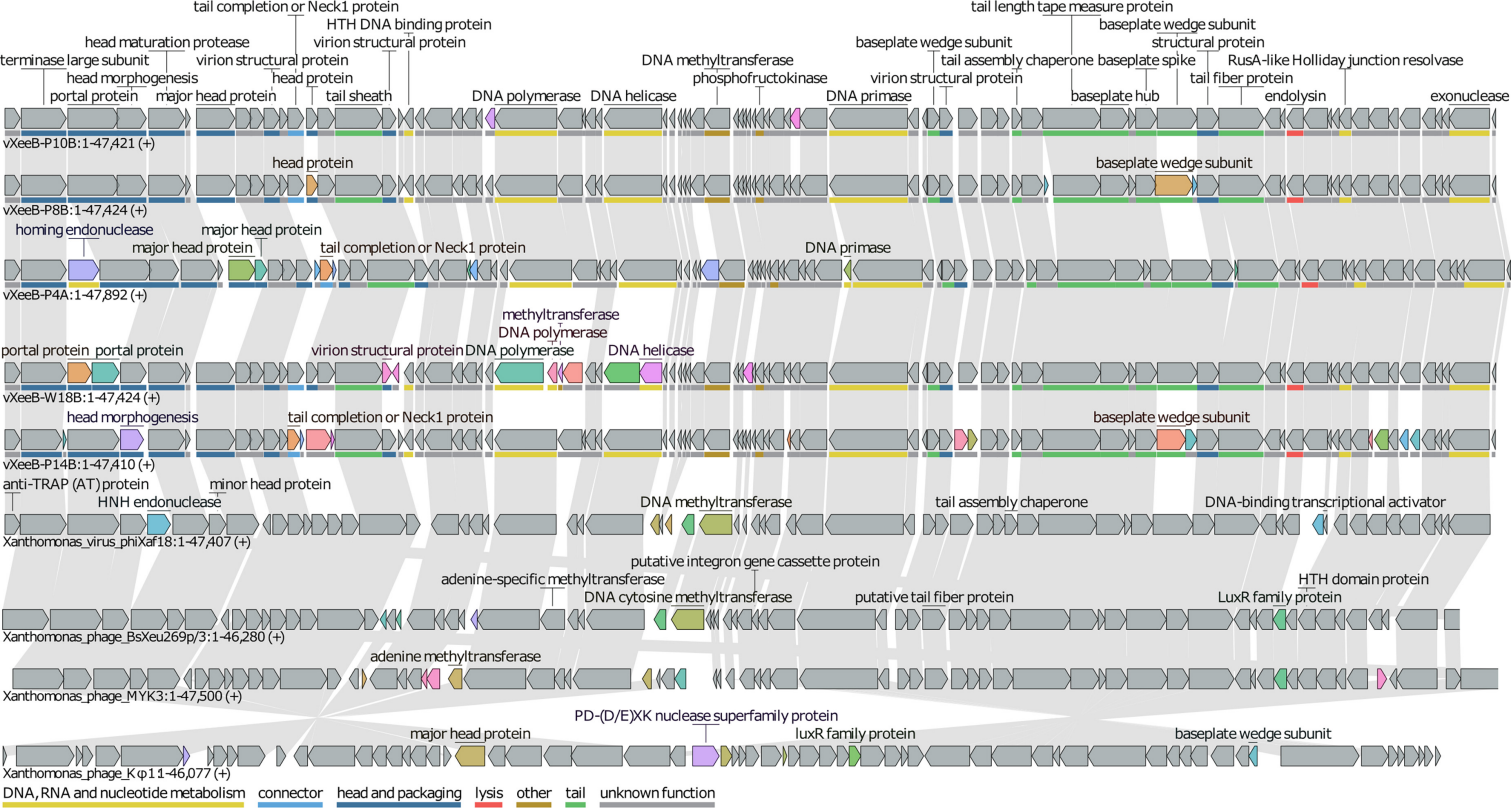
Comparative genome map of *Xanthomonas euvesicatoria* pv. *euvesicatoria* (Xee) phages (vXeeB-P4A, vXeeB-P8B, vXeeB-P10B, vXeeB-P14B, and vXeeB-W18B) and reference phages belonging to the genus *Beograduvirus* and related genera. The alignment was generated using LoVis4u with complete genome sequences. Homologous genomic regions and conserved structural and replication-associated genes are shown along the linearized genomes.

Comparison of genomes revealed a highly conserved genomic organization among the five Xee phages and *Beograduvirus* members (Figure 4). The vXeeB phages shared the same modular arrangement of structural, replication, packaging, and lysis genes observed in *Xanthomonas* phage Kφ1, with no major insertions, deletions, or rearrangements detected. Only vXeeB-P4A exhibited a minor genomic difference corresponding to the insertion of a homing endonuclease gene (nt 2,104–3,063), a typical mobile element in bacteriophages. This insertion accounts for both its slightly larger genome size (47,892 bp) and its lower nucleotide identity (94.7–95.7%) with *Xanthomonas* phages Kφ1 and BsXeu269p/3. Unlike BsXeu269p/3, and phiXaf18, the vXeeB genomes lacked accessory genes such as *luxR*-like regulators or integron-associated elements. Overall, these data confirm the strong genomic conservation within the *Beograduvirus* genus and suggest that vXeeB-P8B, P10B, P14B, and W18B belong to the same species as *Beograduvirus* Kφ1, whereas vXeeB-P4A represents a closely related but a potential new distinct lineage.

Given their high genomic similarity, absence of lysogeny-, virulence-, or antimicrobial resistance–related genes, and the conservation of structural and lytic modules, the five selected vXeeB phages can be considered genetically safe and suitable candidates for biocontrol application against Xee. The lack of genomic elements typically associated with temperate lifestyles reinforces their strictly lytic nature, a key requirement for phage-based biocontrol strategies. Moreover, the high sequence conservation among the isolates from different geographical origins suggests functional stability within this lineage, supporting their potential use as a robust phage cocktail targeting bacterial spot pathogens.

Given the strong conservation of the packaging module across the vXeeB genomes and other *Beograduvirus* members, the DNA packaging strategy of this lineage was further investigated. To date, the DNA packaging mechanism of the *Beograduvirus* genus has not been experimentally characterized. In this study, results provide the first genomic evidence addressing this aspect. *TerL*-base phylogeny analysis grouped the *Beograduvirus* phages in a well-supported monophyletic clade closely related to headful-packaging reference phages (Supplementary Figure 1), and PhageTerm analyses did not identify terminal repeats or cohesive ends using either the PhageTerm or the Li method. Together, these independent lines of evidence strongly support a headful (pac-type) DNA packaging strategy for this group. Interestingly, some *Beograduvirus* genomes deposited in GenBank, such as *Xanthomonas* phage BsXeu269p/3 (ON996340.1) and *Xanthomonas* phage MYK3 (OK275494.1), are reported as circular molecules. This configuration most likely reflects circular permutation and terminal redundancy characteristic of headful-packaging phages, rather than true circular genomes, highlighting the need for careful interpretation of assembly-derived genome topology.

### 3.5 Selected phages lyse a broad panel of *X. euvesicatoria* pv. *euvesicatoria* strains and display high host specificity

**FIGURE 5:**
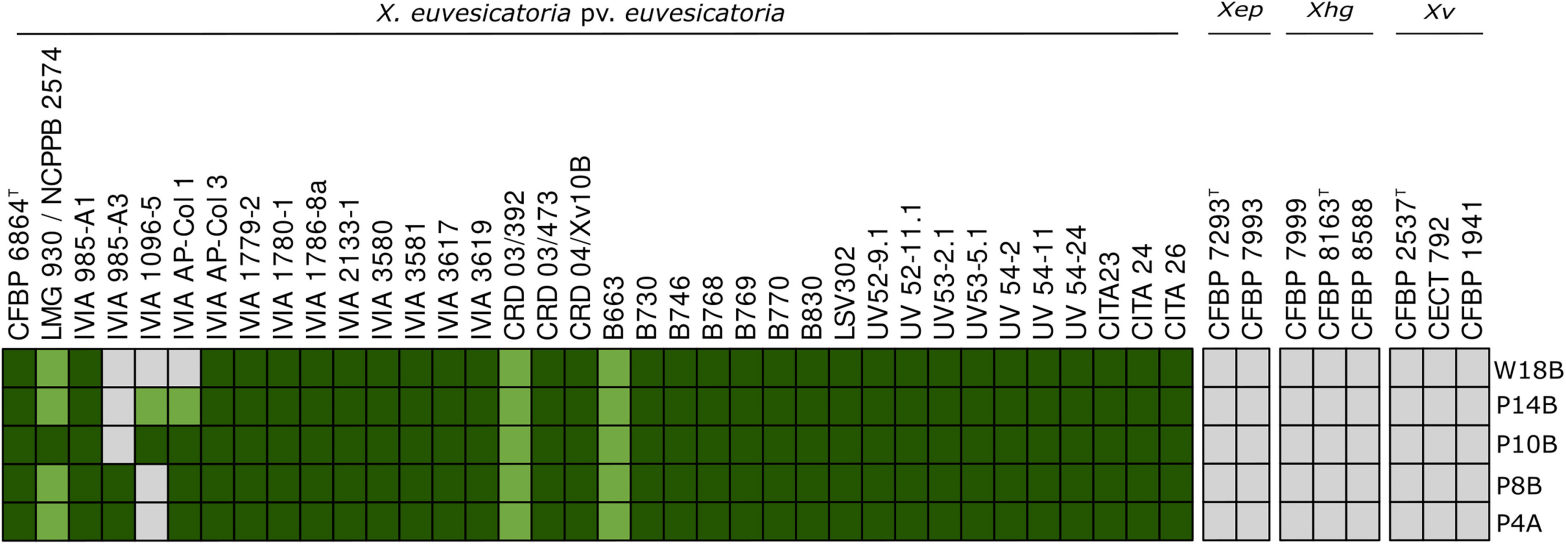
Host range and specificity of bacteriophages infecting *Xanthomonas euvesicatoria* pv. *euvesicatoria* (Xee) and related *Xanthomonas* species. Phage susceptibility was evaluated against a collection of Xee strains, as well as strains belonging to *X. euvesicatoria* pv. *perforans* (Xep), *X. hortorum* pv. *gardneri* and *X. vesicatoria* (Xv). Dark green squares indicate clear lysis, light green squares indicate weak lysis, and grey squares indicate absence of lysis. Phages are shown on the y-axis and bacterial strains on the x-axis (for additional information on phage specificity, see Supplementary Table 1).

Host range analysis revealed that most phages were able to lyse a broad collection of Xee strains from Spain and also the tested strains from the USA. However, differences in lytic profiles were evident among individual phages (Figure 5 and Table 1). Despite this variability, all examined Xee strains, including the type strain, were susceptible to at least two phage isolates, irrespective of the year of isolation, geographical origin, plot or host plant (pepper or tomato) of the strain.

Three out of the five selected Xee phages exhibited clear lytic activity against ≥ 88% of the analyzed strains, while the remaining two were able to lyse a slightly lower percentage (83%) (Figure 5). Based on these results and those of the genomic characterization, phages vXeeB-P4A, P8B, and P10B, which were found to infect a larger number of Xee strains, were selected for further analysis (Table 2). Their bacteriolytic activity was confirmed both in liquid culture and on solid media. Comparative studies across diverse phage groups suggest that variation in receptor-binding proteins, particularly tail and baseplate proteins, can be associated with differences in receptor recognition and host range, supporting the hypothesis that the variability detected among these *Beograduvirus* phages (Figure 4) may contribute to their distinct infectivity patterns (Sun et al., 2023; Ouyang et al., 2024).

High specificity of the Xee phages was further confirmed by the absence of lytic activity against non-target bacteria, including other *Xanthomonas* species that cause bacterial spot in Spain (*X. vesicatoria*) and in other countries (*X. euvesicatoria* pv. *perforans* and *X. hortorum* pv. *gardneri*), as well as other *Xanthomonas* species that are not associated with this disease. No lytic activity was detected against other phytopathogenic bacteria from different genera or against bacterial isolates from the pepper plant-associated microbiota (Supplementary Tables 1, 2).

### 3.6 *X. euvesicatoria* pv. *euvesicatoria* phages display myovirus morphology

**FIGURE 6:**
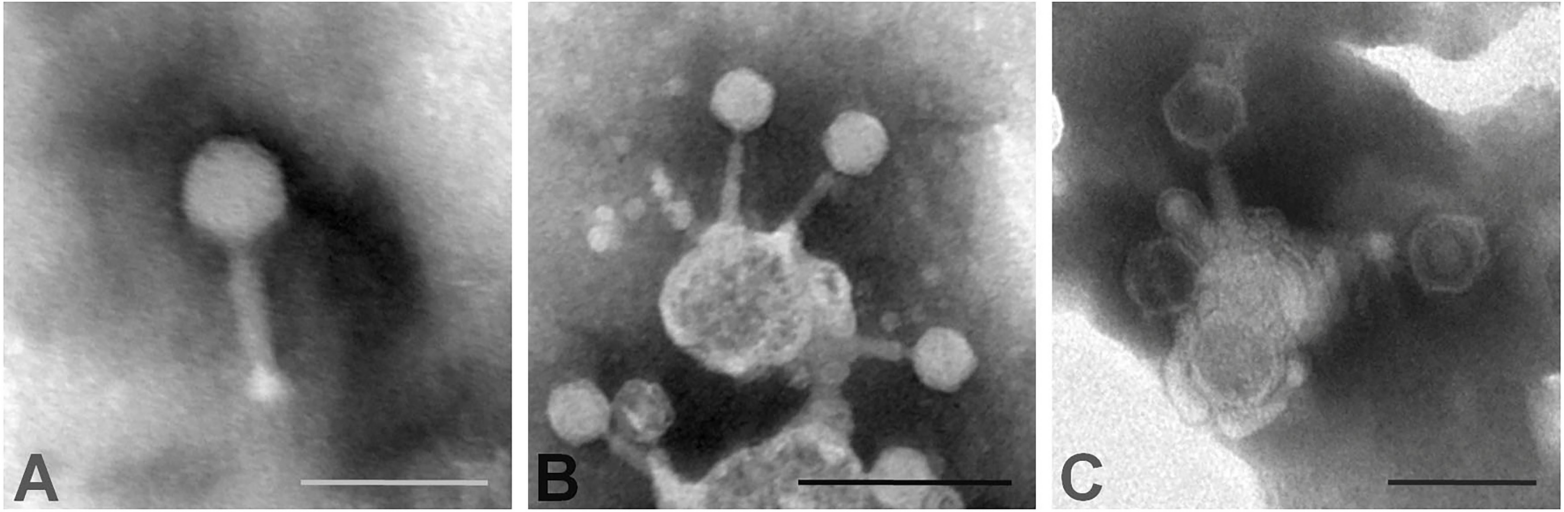
Morphology of negatively stained virions of *Xanthomonas euvesicatoria* pv. *euvesicatoria* (Xee) phages. **(A)** vXeeB-P4A, **(B)** vXeeB-P8B, and **(C)** vXeeB-P10B. Transmission electron micrographs were obtained at x70,000, x80,000 and x60,000 magnification, respectively. The scale bar represents 100 nm in **(A)** and **(C)**, and 200 nm in **(B)**.

Three phages (vXeeB-P4A, P8B, and P10B) were selected for morphological characterization based on the results of the genomic analysis, the host range, and the phage source and origin. According to the transmission electron micrographs obtained, the virions of phages vXeeB-P4A, P8B and P10B were characterized by an icosahedral capsid and a long contractile tail (Figures 6A–C). Therefore, the three phages studied were classified in the present class *Caudoviricetes* (former order *Caudovirales*) based on their morphology (Turner et al., 2023), coinciding with previous studies of lytic phages of Xee (Gašić et al., 2011; Kizheva et al., 2023), showing myovirus morphology. In this work, the average diameter of the capsids for phages vXeeB-P4A, P8B, and P10B was 58.95 ± 2.90 nm, 57.83 ± 3.13 nm, and 57.65 ± 2.13 nm, respectively. The average tail length for phage vXeeB-P4A was 71.05 ± 6.05 nm, for phage P8B 67.71 ± 5.30 nm and for phage P10B 65.16 ± 4.71 nm. The morphology of the three Xee-selected phages is consistent with that previously reported for the Serbian Xee phage Kφ1 and the Bulgarian Xee phage BsXeu269p/3. However, the capsids of the vXeeB-P4A, P8B, and P10B phages are larger than that of the Kφ1 phage, which has a diameter of approximately 51.8 ± 2.5 nm (Gašić et al., 2011), and that of the BsXeu269p/3 phage, which has a diameter of around 50 nm (Kizheva et al., 2023). The respective lengths of their tails are approximately 79.0 ± 9.0 nm and 100 nm.

### 3.7 High stability of *X. euvesicatoria* pv. *euvesicatoria* phages across the tested temperature and pH conditions

**FIGURE 7:**
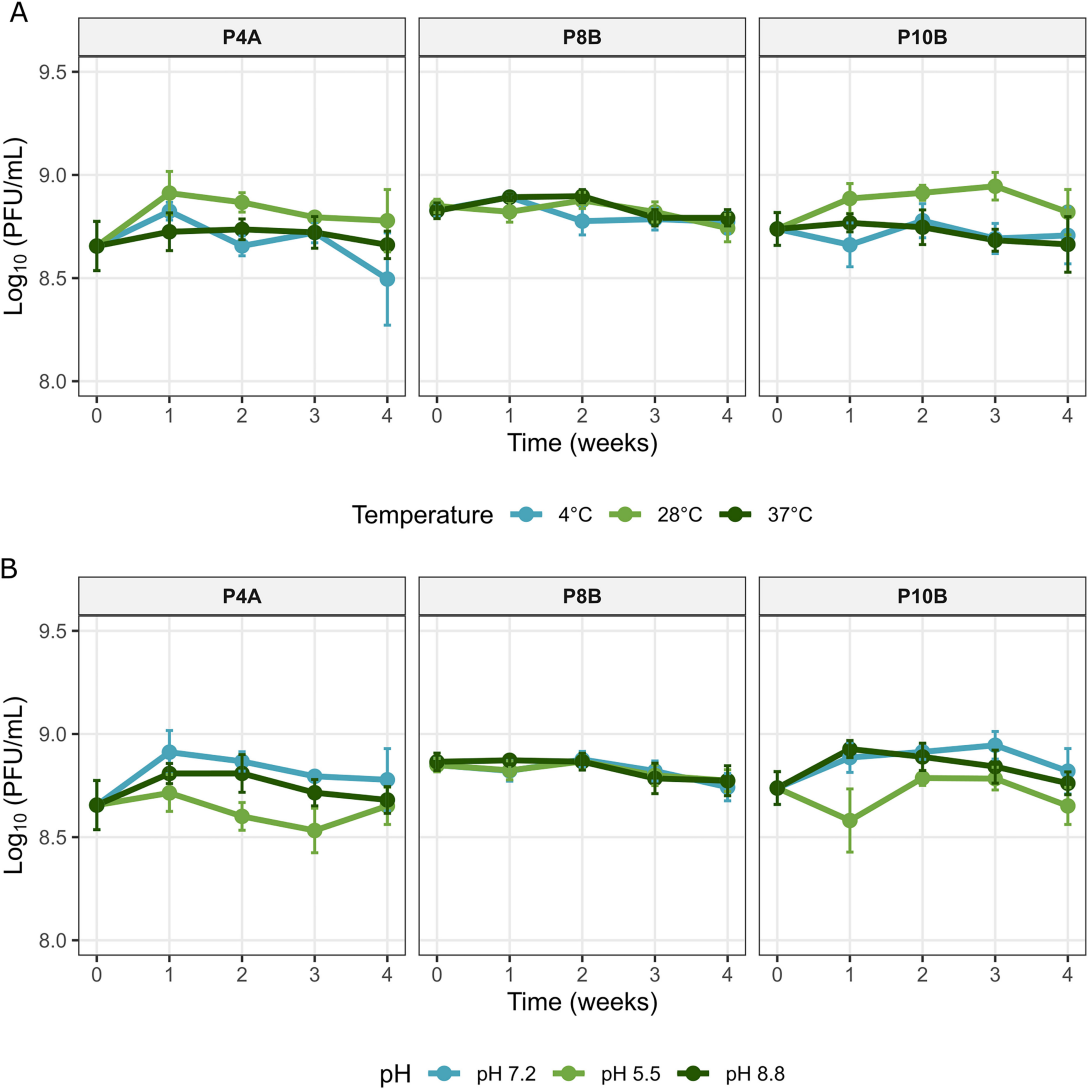
Survival of *Xanthomonas euvesicatoria* pv. *euvesicatoria* (Xee) phages. Survival of vXeeB-P4A (P4A), vXeeB-P8B (P8B) and vXeeB-P10B (P10B) at different temperatures **(A)**, and pH values **(B)** in SM buffer. Phage survival was assessed by determining PFU/mL at 0, 1, 2, 3, and 4 weeks of incubation under each condition. All assays were conducted in duplicate with triplicate plaque counts per sample. Data are presented as mean PFU/mL ± standard deviation.

The environmental stability of phages is a critical parameter for the development of effective phage-based biocontrol strategies, as it determines their persistence and potential efficacy in agricultural settings. Xee phages must be able to maintain their infectivity in the face of the pH and temperature fluctuations that are characteristic of the phyllosphere. The three phages described in the present study have a particularly noteworthy characteristic: they maintain titers close to the initial ones (≈10^8^ PFU/mL) for at least one month in SM buffer under a temperature range of 4–37°C and three pH values between 5.5 and 8 (Figures 7A,B). Linear mixed-effect models supported this observation, revealing no significant influence of temperature or pH on phage survival (*p* > 0.05) in the assayed conditions. This result indicates a high and comparable stability of the three phages and represents a substantial improvement over previous studies (Gašić et al., 2011, 2018; Shopova et al., 2023).

The first stability analysis of Xee phages was conducted by Gašić et al. (2011), focused mainly on the extreme heat tolerance of several phages isolated in Serbia. The authors evaluated thermal inactivation by incubating phage suspensions of 10^7^ PFU/mL for 10 min at a gradient of 35–75°C. The results showed that, although all phages exhibited some degree of temperature tolerance, they were inactivated at 70°C. However, the study did not provide information on their stability over prolonged periods under more realistic environmental conditions. Subsequently, Gašić et al. (2018) expanded the survival study of the Serbian Xee phage Kφ1, evaluating several relevant environmental factors, such as pH values. In pH tests, the Kφ1 phage remained stable after 24 h in the pH range of 5–11, while it was inactivated at extreme values (pH 2 and 12). More recently, Shopova et al. (2023) demonstrated that the Bulgarian Xee BsXeu269p/3 phage exhibited significant stability in the phyllosphere. However, a sharp decline was observed in soil microcosms, with a ∼8 log decrease in titer over ∼55 days. In this context, titers close to the initial ones (about 10^8^ PFU/mL) were maintained by the three Xee phages described in this study for at least one month in SM buffer under three pH values between 5.5 and 8, as well as in a temperature range of 4–37°C. These data improve those obtained in previous studies, as with the Kφ1 phage (Gašić et al., 2018), where pH stability only was evaluated at 24 h, and as with the phage BsXeu269p/3 (Shopova et al., 2023), where the stability was evaluated at 48 h. There was no equivalent data on prolonged maintenance at moderate temperatures. As the pH and temperature ranges tested in this study closely match typical leaf surface in the conditions under which the phages were isolated, these Xee phages are promising candidates for biocontrol applications.

Overall, the comparative results suggest that, while Serbian and Bulgarian Xee phages exhibit adequate stability under moderate conditions, the phages selected in this study are notable for their sustained stability over weeks within realistic environmental ranges.

### 3.8 The three selected phages control *X. euvesicatoria* pv. *euvesicatoria* in co-culture, individually and combined

**FIGURE 8:**
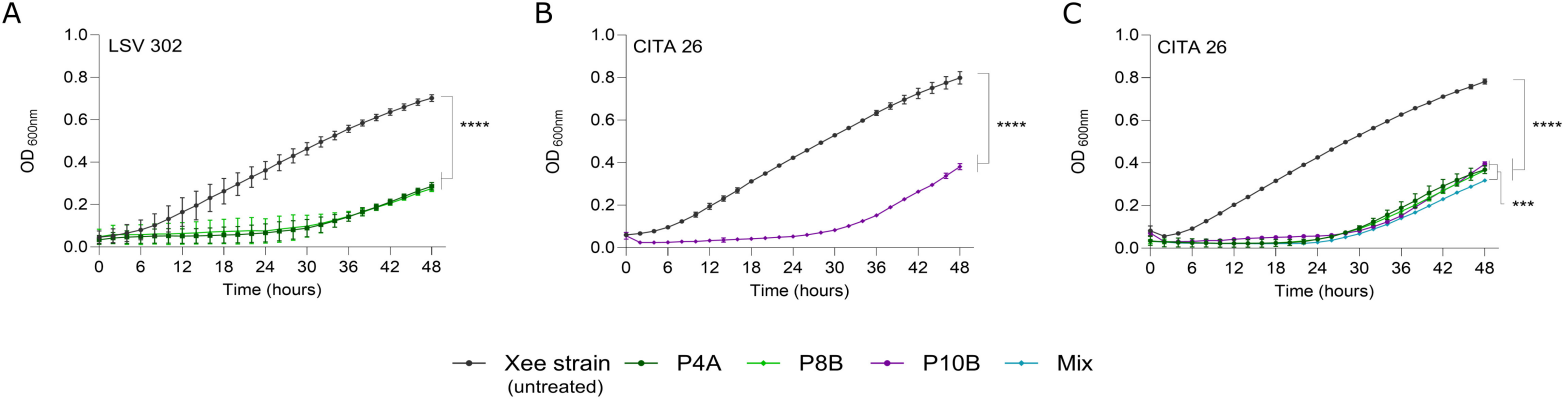
*In vitro* biocontrol activity of bacteriophages against *Xanthomonas euvesicatoria* pv. *euvesicatoria* (Xee). Growth dynamics of Xee strains LSV 302 **(A)** and CITA 26 **(B,C)** at a multiplicity of infection (MOI) of 10 in LB medium, either alone (untreated control) or in co-culture with phages vXeeB-P4A (P4A), vXeeB-P8B (P8B), and vXeeB-P10B (P10B), tested individually **(A,B)** or combined as a three-phage cocktail *versus* single phages **(C)**, during 48 h of incubation at 28°C. Data represent the mean mostly with six replicates, and error bars indicate standard deviation. Area under the curve (AUC) values were used to quantify overall bacterial growth. In all cases, phage treatments significantly reduced bacterial growth compared to the untreated control (*p* < 0.0001). No significant differences were detected among individual phage treatments (Bonferroni-adjusted *p* > 0.05). In contrast, the three-phage cocktail resulted in a significantly greater reduction in bacterial growth compared to single-phage treatments (*p* < 0.001). Statistical significance is indicated by asterisks (***p < 0.001; ****p < 0.0001; Bonferroni-adjusted comparisons).

The ability of the three selected Xee phages to exert biocontrol of the pathogen in liquid co-culture was first evaluated by monitoring the dynamics of the individual phages at a MOI of 10 (bacterium 10^7^ CFU/mL: phage 10^8^ PFU/mL), using the LSV 302 or CITA 26 strains from Extremadura and Aragón, respectively, as the host strain. When the individual phages were tested against their respective host strain, all phages significantly reduced bacterial growth compared to the bacterial untreated control, as indicated by the lower area under the curve (AUC) values (Figures 8A,B). An additional comparative test was carried out in which the three-phage cocktail and the phages individually were tested against strain CITA 26 simultaneously at a MOI of 10. Consistent with the previous assays, no significant differences were detected among the individual phage treatments. However, the phage cocktail was associated with a significantly lower area under the growth curve compared with the untreated control and the co-culture with single phages, as determined by statistical analysis (Figure 8C). In line with the growth curves, the OD_600_ values remained close to the baseline for approximately the first 30 h of the 48-h incubation period, followed by a gradual increase in optical density likely associated with the emergence of resistant bacterial subpopulations, even in the presence of the phage cocktail. Nevertheless, bacterial densities at the end of the experiment remained significantly lower (OD_600_ 0.3-0.4) than those of the untreated controls (OD_600_ 0.7–0.8) (Figure 8). Similar results are not available for the phages Xee Kφ1 and BsXeu269p/3 but, phages described for other *Xanthomonas* species affecting different horticultural crops, such as *X. campestris*, have been studied in the context of biological control. In this regard, the FoX4 phage has demonstrated reduced lytic activity over a shorter period of time, albeit at lower MOIs (0.1 and 1) (Holtappels et al., 2022). Thus, the selected Xee phages demonstrate significant lytic activity that persists for at least 48 h. This is important because, under *in vivo* conditions, apart from phages, bacterial pathogens have to cope with competitor plant microbiota and plant defenses (Álvarez et al., 2019).

The dynamics of bacterium-phage interactions in the *in vitro* biocontrol of Xee has not been the focus of previous publications (Gašić et al., 2011, 2018; Shopova et al., 2023). However, studies on other phytopathogenic bacterial species of *Xanthomonas*, as well as species from other genera, such as *E. amylovora* and *Ralstonia solanacearum*, have demonstrated the superiority of phage cocktails over single phages in reducing the growth of the target pathogens (Álvarez et al., 2019; Domingo-Calap et al., 2022; Biosca et al., 2024).

### 3.9 *X. euvesicatoria* pv. *euvesicatoria* phage cocktail effectively controls bacterial spot in pepper plants

**FIGURE 9:**
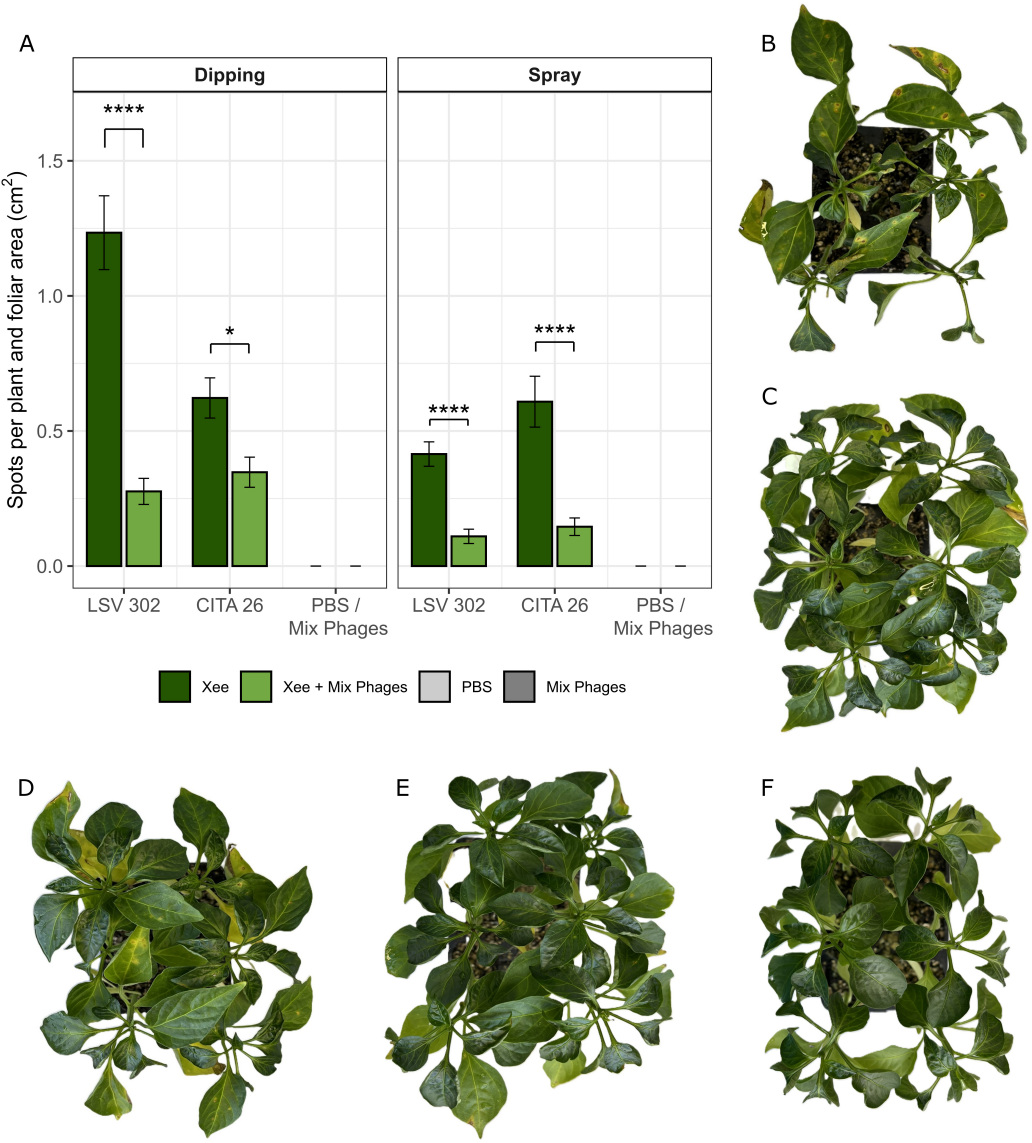
Phage-based biocontrol of *Xanthomonas euvesicatoria* pv. *euvesicatoria* (Xee). Biocontrol assays of Xee strains LSV 302 and CITA 26 in pepper plants using a cocktail of the phages vXeeB-P4A, vXeeB-P8B, and vXeeB-P10B. **(A)** Lesion counts normalized by foliar area (mean ± SD; n = 18 plants per condition, corresponding to three independent experiments) after inoculation by leaf dipping or foliar spraying. Statistical significance is indicated by asterisks (*p < 0.05; ****p < 0.0001; Bonferroni-adjusted). **(B–F)** Representative photographs of pepper plants at 21 days post-inoculation: positive control **(B)** and phage treatment by dipping **(C)** for strain LSV 302; positive control **(D)** and phage treatment by dipping **(E)** for strain CITA 26; negative control inoculated with PBS **(F).**

Based on the *in vitro* co-culture results, the biocontrol potential of the three selected lytic Xee phages, combined in a cocktail, was evaluated in pepper plants through co-inoculation with Xee strains LSV 302 and CITA 26 at a MOI of 10 (Xee at 10^7^ CFU/mL: phages at 10^8^ PFU/mL). This MOI has been shown to result in significant and sustained inhibition of bacterial growth in previous studies (Biosca et al., 2024). This evaluation included the corresponding positive controls (LSV 302 or CITA 26 alone) and negative controls (PBS or the phage cocktail alone) for the disease. Plants were co-inoculated with bacteria and phages using two application methods: leaf dipping and foliar spraying. Disease severity was assessed at 21 dpi by quantifying the lesion number per plant and normalizing values to leaf surface area. Negative control plants inoculated with PBS or with the phage cocktail alone remained symptom-free. Data are presented as mean ± standard deviation from three independent experiments (Figure 9A).

A three-way ANOVA (Type III) on log-transformed lesion data showed that phage treatment significantly reduced disease severity overall (*p* = 0.012), although this effect was not uniform across the experimental conditions. Significant two-way interactions revealed that the magnitude of disease reduction was modulated by the application method (*p* = 0.0048) and the bacterial strain (*p* = 0.011). In addition, disease development in response to each inoculation method was strain-specific, as dipping and spraying did not produce equivalent disease levels for LSV 302 and CITA 26 (method × strain, *p* = 0.0018). These results highlight strain-dependent differences in disease progression and treatment response. The three-way interaction (treatment × method × strain) was non-significant (*p* = 0.051).

*Post hoc* comparisons (Bonferroni-adjusted) clarified the biological manifestation of these interactions. For strain LSV 302, both dipping and spraying with phages produced similarly strong reductions in lesion development (77.6 and 73.5%, respectively, both *p* < 0.0001). However, for strain CITA 26, phage application via foliar spraying resulted in a substantially greater reduction (76.1%, *p* < 0.0001) than dipping (44.2%, *p* = 0.0123). Overall, foliar spraying of the phage cocktail provided the most consistent and significant reductions across strains, representing a practical application method for greenhouse and field use.

Representative images of biocontrol assays by dipping are shown in Figures 9B–E, with PBS controls in Figure 9F. Consistent with lesion severity results, positive control plants exhibited reduced leaf numbers compared to phage-treated plants. Representative photographs corresponding to biocontrol test by foliar spraying are provided in Supplementary Figure 2.

Previous studies have evaluated the efficacy of different Xee phages, either applied individually or according to different protocols, against Xee strains from two Southeastern European countries. Gašić et al. (2018) sprayed phage Kφ1, isolated from the rhizosphere of a pepper plant affected by bacterial spot, onto pepper leaves at a MOI of 1 against a Serbian Xee strain. Treatments included single or double phage applications by spraying, either 2 h before or 15 min after bacterial inoculation, as well as a combined treatment with copper hydroxide. After 3 weeks, phage application resulted in a significant reduction of approximately 30 and 50% following single and double applications, respectively, in the number of bacterial spot lesions on five leaves per plant. However, the number of plants included in each experiment was not specified. Furthermore, no significant difference was found in disease reduction between phage applications before and after pathogen inoculation. Authors also concluded that a single application of phage Kφ1 was not consistently effective and that the better treatment was a combination of phage and copper.

More recently, Shopova et al. (2023) tested the efficacy of a single phage, BsXeu269p/3, which was isolated from the rhizosphere of tomato plants affected by bacterial spot against a Bulgarian strain of the pathogen. The authors applied the phage at a MOI of 10 by needle injection to half of the leaves and by spraying 2 and 8 days after inoculation, in trials lasting 11 and 14 days respectively, to evaluate the effect of the phage treatment on horizontal bacterial transmission from infected to healthy plants. The activity of BsXeu269p/3 was assessed based on a reduction in pathogen populations, as determined by quantitative PCR (qPCR), rather than a reduction in disease symptoms. Spray treatment reduced the number of Xee on the leaf surface by a factor of five, using four plants per pot and three pots per set of tested conditions. Injection treatment significantly reduced the pathogen in treated lesions by around 60%, compared to the untreated group, but the number of plants used in each experiment was not specified. It should be noted that direct comparisons between the present study and previous ones are limited due to the many differences in the biocontrol protocols used. According to Shopova et al. (2023), where a similar MOI was employed via spraying, the effect of the phage was quantified by reductions in bacterial populations rather than reductions in symptoms. Although these parameters are related, they reflect different aspects of the infection process. Nevertheless, the spot reductions observed in this work (44.2–77.6%) are at the upper end of the previously reported range, potentially due to the use of a phage cocktail rather than a single phage. Bacteriophage cocktails have been shown to enhance biocontrol efficacy in plants and reduce the likelihood of resistance development compared to single-phage treatments (Álvarez et al., 2019; Nakayinga et al., 2021; Biosca et al., 2024). Furthermore, the results of this study are highly promising, demonstrating the effectiveness of a single treatment with the three-phage cocktail.

Overall, in this work, a collection of phages was isolated from pepper plots experiencing bacterial spot outbreaks in two distant regions of Spain. Genomic analysis of five selected of them confirmed they belonged to the *Beograduvirus* genus, and were lytic and safe. Three of the phages were further characterized due to their ability to infect multiple Xee strains while showing high specificity. These phages have myovirus morphology and are stable at temperatures and pH values that mimic field conditions. They have also been shown to significantly reduce pathogen growth *in vitro* when used combined in a cocktail. This three-phage cocktail has also been proved to be highly effective in reducing disease symptoms in pepper plants co-inoculated with the pathogen by foliar dipping or spraying. This is the first European *Xanthomonas euvesicatoria* pv. *euvesicatoria* phage cocktail effective in controlling bacterial spot in pepper plants, offering a promising strategy for integrated disease management in line with the One Health approach and the Sustainable Development Goals.

## Data Availability

The genome sequences of the five Mediterranean *Xanthomonas euvesicatoria* pv. *euvesicatoria* phages (vXeeB-P4A, vXeeB-P8B, vXeeB-P10B, vXeeB-P14B, and vXeeB-W18B) were deposited at GenBank (PZ019799, PZ019800, PZ019801, PZ019802, and PZ019803).

## Footnotes

1
https://github.com/nanoporetech/dorado
