# Supplementary material for: Development of the first European Xanthomonas euvesicatoria pv. euvesicatoria lytic bacteriophage cocktail effective in controlling bacterial spot disease in pepper plants
Source: Front Microbiol. 2026 May 7;17:1821339. doi: 10.3389/fmicb.2026.1821339 (PMC13190192; doi:10.3389/fmicb.2026.1821339)
Supplement: Supplementary file 1 [file Data_Sheet_1.docx]

Development of the first European *Xanthomonas euvesicatoria* pv. *euvesicatoria* lytic bacteriophage cocktail effective in controlling bacterial spot disease in pepper plants

Elena G. Biosca^1*^ ^†^, Isabel Salas-Lastres^1 †^, José Francisco Català-Senent^1^, Félix Morán^1**^, Ana Palacio-Bielsa^2,3^, Belén Álvarez^1,4^

^1^Departamento de Microbiología y Ecología, Universitat de València, Valencia, Spain

^2^Departamento de Sistemas Agrícolas, Forestales y Medio Ambiente, Centro de Investigación y Tecnología Agroalimentaria de Aragón (CITA), Zaragoza, Spain

^3^Instituto Agroalimentario de Aragón – IA2 (CITA-Universidad de Zaragoza), Zaragoza, Spain

^4^Área de Investigación Agroambiental, Instituto Madrileño de Investigación y Desarrollo Rural, Agrario y Alimentario (IMIDRA), Madrid, Spain

***** Correspondence: [elena.biosca@uv.es](mailto:elena.biosca@uv.es)

† First authorship: These authors share first authorship.

**Present address: Centro de Protección Vegetal y Biotecnología, Instituto Valenciano de Investigaciones Agrarias (IVIA), Valencia, Spain.

Supplementary Material

# Supplementary Tables

**Supplementary Table 1.** Bacterial species, including other phytopathogens and pepper plant microbiota, used for *Xanthomonas euvesicatoria* pv*. euvesicatoria* phages specificity assays.

| **Bacterial Species** | **Strain** | **Source** | **Country** |
| --- | --- | --- | --- |
| ***Phytopathogens*** | | | |
| *Burkholderia gladioli* | CECT^1^ 4148^T^ | *Gladiolus* sp. | USA |
| *Burkholderia glumae* | LMG^2^ 2196^T^ | *Oryza sativa* | Japan |
| *Clavibacter michiganensis* | IVIA^3^ 873 | *Solanum lycopersicum* | Spain |
| *Dickeya* sp. | IVIA 4830 | *Solanum lycopersicum* | Spain |
| *Erwinia amylovora* | IVIA 1554 | *Crataegus* sp. | Spain |
| *Pectobacterium atrosepticum* | IVIA 3447 | *Solanum tuberosum* | Spain |
| *Pectobacterium carotovorum* | IVIA 3902 | *Solanum lycopersicum* | Spain |
| *Pseudomonas savastanoi* pv. *savastanoi* | IVIA 1628.3 | *Olea europaea* | Spain |
| *Rhizobium radiobacter* | C58 | *Prunus avium* | USA |
| *Rhizobium rhizogenes* | K84 | Non-pathogenic | Australia |
| *Ralstonia solanacearum* | IVIA 1670 | *Solanum tuberosum* | Spain |
| *Xanthomonas arboricola* pv. *pruni* | CITA^4^ 33 | *Prunus amygdalus* | Spain |
| ***Pepper plant microbiota*** | | | |
| *Pseudomonas* sp. | UV^5^-5 | *Capsicum annuum* | Spain |
| *Microbacterium* sp. | UV-6 | *Capsicum annuum* | Spain |
| *Microbacterium* sp. | UV-7 | *Capsicum annuum* | Spain |
| *Microbacterium* sp. | UV- 9 | *Capsicum annuum* | Spain |
| *Microbacterium* sp. | UV-10 | *Capsicum annuum* | Spain |
| *Arthrobacter* sp. | UV-14 | *Capsicum annuum* | Spain |
| *Microbacterium* sp. | UV-15 | *Capsicum annuum* | Spain |
| *Pseudomonas* sp. | UV-16 | *Capsicum annuum* | Spain |
| *Pseudoclavibacter sp.* | UV-18 | *Capsicum annuum* | Spain |
| *Pseudomonas* sp. | UV-21 | *Capsicum annuum* | Spain |
| ***Strains from different environments*** | | | |
| *Aeromona shydrophila* | CECT 5173 | Freshwater | France |
| *Alcaligenes faecalis* | CECT 928 | Unknown | Unknown |
| *Bacillus cereus* | CECT 495 | Chicken and turkey manure | Unknown |
| *Enterococcus faecalis* | CECT 481 | Unknown | Unknown |
| *Escherichia coli* | CECT 101 | Unknown | United Kingdom |
| *Klebsiella pneumonia* | CECT 143 | Unknown | United States |
| *Kocuria rhizophila* | CECT 241 | Soil | Unknown |
| *Pseudomonas fluorescens* | CECT 378 | Pre-filter tanks, town water works | United Kingdom |
| *Proteus hauseri* | CECT 484 | Unknown | Unknown |
| *Salmonella enterica* subsp. *enterica* | CECT 443 | Human food poisoning | United Kingdom |
| **Bacterial Species** | **Strain** | **Source** | **Country** |
| *Serratia marcescens* | CECT 159 | Unknown | Unknown |
| *Staphylococcus aureus* | CECT 4013 | Bovine mammary gland | Unknown |

**^1^ CECT**: Colección Española de Cultivos Tipo (Spanish Type Culture Collection); ^2^**LMG**: Belgian Coordinated Collections of Microorganisms: Bacteria Collection; **^3^IVIA**: Instituto Valenciano de Investigaciones Agrarias; **^4^ CITA**: Centro de Investigación y Tecnología Agroalimentaria de Aragón; and **^5^ UV**: Universitat de València; **T**, Type strain.

**Supplementary Table 2.** Host range and specificity of *Xanthomonas euvesicatoria* pv*. euvesicatoria* phages.

| **Host strain code** | **UV X. euvesicatoria bacteriophages** | | | | |
| --- | --- | --- | --- | --- | --- |
|  | **P4A** | **P8B** | **P10B** | **P14A** | **W18B** |
| **Other phytopathogens** | | | | | |
| Burkholderia gladioli | - | - | - | - | - |
| Burkholderia glumae | - | - | - | - | - |
| Clavibacter michiganensis | - | - | - | - | - |
| Dickeya chrysanthemi. | - | - | - | - | - |
| Pseudomonas syringae pv. syringae | - | - | - | - | - |
| Pseudomonas savastanoi pv. savastanoi | - | - | - | - | - |
| Rhizobium radiobacter | - | - | - | - | - |
| Rhizobium rhizogenes | - | - | - | - | - |
| Ralstonia solanacearum | - | - | - | - | - |
| Xanthomonas arboricola pv. pruni | - | - | - | - | - |
| **Pepper associated-bacteria** | | | | | |
| Pseudomonas sp. | - | - | - | - | - |
| Microbacterium sp. | - | - | - | - | - |
| Microbacterium sp. | - | - | - | - | - |
| Microbacterium sp. | - | - | - | - | - |
| Microbacterium sp. | - | - | - | - | - |
| Arthrobacter sp. | - | - | - | - | - |
| Microbacterium sp. | - | - | - | - | - |
| Pseudomonas sp. | - | - | - | - | - |
| Pseudoclavibacter sp. | - | - | - | - | - |
| Pseudomonas sp. | - | - | - | - | - |
| **Strains from different environments** | | | | | |
| Aeromonas hydrophila | - | - | - | - | - |
| Alcaligenes faecalis | - | - | - | - | - |
| Bacillus cereus | - | - | - | - | - |
| Enterobacter cloacae | - | - | - | - | - |
| Enterococcus faecalis | - | - | - | - | - |
| Escherichia coli | - | - | - | - | - |
| Klebsiella pneumoniae | - | - | - | - | - |
| Klebsiella rhizophila | - | - | - | - | - |
| Pseudomonas fluorescens | - | - | - | - | - |
| Proteus vulgaris | - | - | - | - | - |
| Salmonella entérica | - | - | - | - | - |
| Serratia marcescens | - | - | - | - | - |
| Staphylococcus aureus | - | - | - | - | - |

-, no clearing zone observed in spot assay.

#
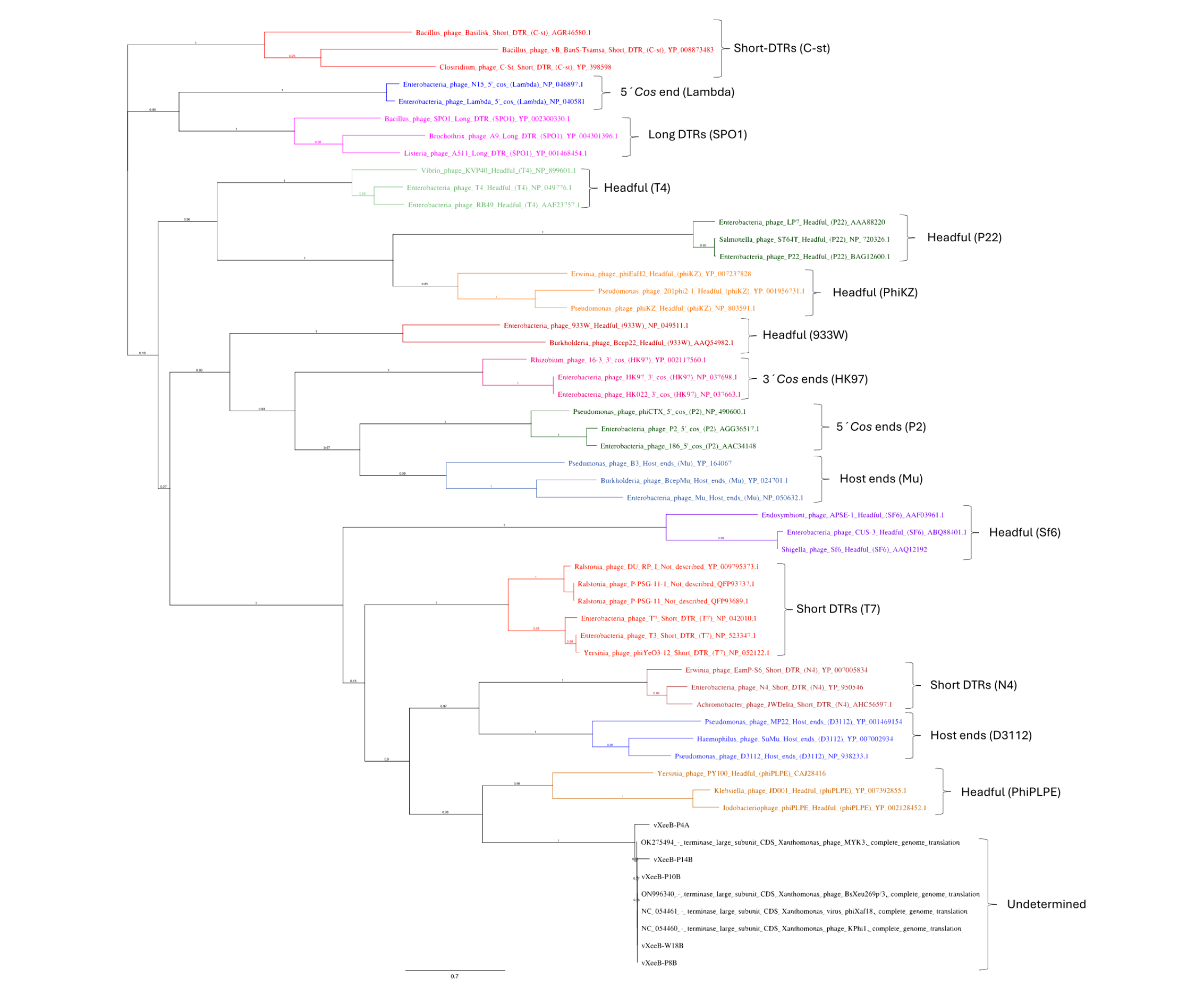
Supplementary Figures

**Supplementary Figure 1.** Phylogenetic tree of the large terminase subunit (*terL*) proteins of the five *Xanthomonas euvesicatoria* pv. *euvesicatoria* phages vXeeB-P4A, vXeeB-P8B, vXeeB-P10B, vXeeB-P14B and vXeeB-W18B (in bold and italics) and 50 reference phages representing major DNA packaging types. Sequences were aligned with Clustal Omega, and the tree was inferred using FastTree v2.1.11 implemented in Geneious Prime. Packaging groups are colour-coded.

**
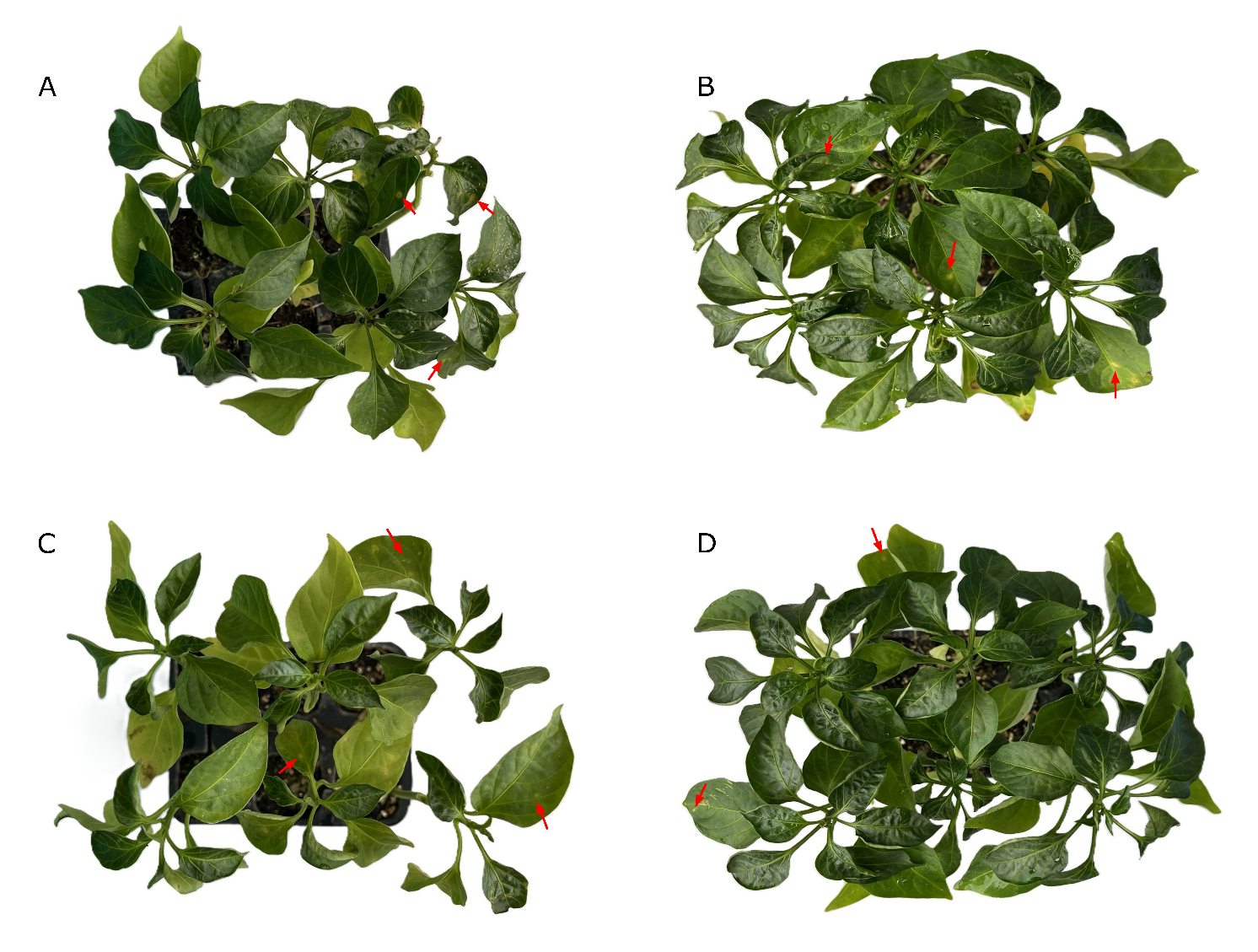
**

**Supplementary Figure 2.** Representative photographs of pepper plants at 21 days post-inoculation: positive control **(A)** and phage treatment by spray **(B)** for *Xanthomonas euvesicatoria* pv. *euvesicatoria* (Xee) strain LSV 302; positive control **(C)** and phage treatment by spray **(D)** for Xee strain CITA 26. Red arrows indicate typical lesions.
